# The MYB33, MYB65, and MYB101 transcription factors affect *Arabidopsis* and potato responses to drought by regulating the ABA signaling pathway

**DOI:** 10.1111/ppl.13775

**Published:** 2022-09-26

**Authors:** Anna Wyrzykowska, Dawid Bielewicz, Patrycja Plewka, Dorota Sołtys‐Kalina, Iwona Wasilewicz‐Flis, Waldemar Marczewski, Artur Jarmolowski, Zofia Szweykowska‐Kulinska

**Affiliations:** ^1^ Department of Gene Expression, Institute of Molecular Biology and Biotechnology, Faculty of Biology Adam Mickiewicz University Poznań Wielkopolskie Poland; ^2^ Plant Breeding and Acclimatization Institute – National Research Institute Młochów Masovian Voivodeship Poland

## Abstract

Drought is one of the main climate threats limiting crop production. Potato is one of the four most important food crop species worldwide and is sensitive to water shortage. The *CBP80* gene was shown to affect *Arabidopsis* and potato responses to drought by regulating the level of microRNA159 and, consequently, the levels of the MYB33 and MYB101 transcription factors (TFs). Here, we show that three MYB TFs, MYB33, MYB65, and MYB101, are involved in plant responses to water shortage. Their downregulation in *Arabidopsis* causes stomatal hyposensitivity to abscisic acid (ABA), leading to reduced tolerance to drought. Transgenic *Arabidopsis* and potato plants overexpressing these genes, with a mutated recognition site in miR159, show hypersensitivity to ABA and relatively high tolerance to drought conditions. Thus, the *MYB33*, *MYB65*, and *MYB101* genes may be potential targets for innovative breeding to obtain crops with relatively high tolerance to drought.

## 1 INTRODUCTION

Drought is one of the most visible effects of climate change currently worldwide. These effects are also some of the most drastic factors affecting agriculture and the economy. Via classic and molecular approaches, many genomic loci and genes potentially associated with the response to water shortage have been identified in model and crop species (Chaves et al., 2003; Duque et al., 2013; Joshi et al., 2016; Kulkarni et al., 2017). Potato (*Solanum tuberosum* L.) is considered a drought‐sensitive crop species, although cultivar‐dependent differences in tolerance have been described (Boguszewska‐Mańskowska et al., 2018; Dahal et al., 2019; Gervais et al., 2021; Lehretz et al., 2021; Pieczynski et al., 2017; Soltys‐Kalina et al., 2016; Zarzyńska et al., 2017). Tolerance to drought is a very complex polygenic trait in potato (Obidiegwu et al., 2015). Several genes, as well as quantitative trait loci, for drought tolerance have been identified on all 12 potato chromosomes (Anithakumari et al., 2011, 2012; Khan et al., 2015; Pieczynski et al., 2018).

Abscisic acid (ABA) is a phytohormone produced in response to abiotic stress, including drought stress (Lee & Luan, 2012). ABA acts at the cellular level by inducing stomatal closure and inducing/repressing stress‐related genes. DREB2A/2B, AREB1RD22BP1 and MYC/MYB represent the main group of TFs that regulate ABA‐responsive gene expression. These proteins bind to their corresponding *cis*‐acting elements within promoter regions, such as Dehydration ‐responsive elements/C‐repeats, ABA‐ responsive elements (ABRE) and MYC recognition sequence/ MYB recognition sequence (MYCRS/MYBRS) (Fujita et al., 2011; Nakashima & Yamaguchi‐Shinozaki, 2013; Nishimura et al., 2009; Wang et al., 2011).

Genes involved in RNA metabolism have been found to affect the ABA signaling pathway (Hugouvieux et al., 2001, 2002; Laloum et al., 2018; Pieczynski et al., 2013; Reyes & Chua, 2007; Roychoudhury et al., 2013). For example, it was found that alternative splicing, affected by Serine and arginine‐ rich  proteins, can produce three alternative protein isoforms of the zinc‐induced‐facilitator‐like 1 (ZIFL1) transporter required for polar auxin transport in *Arabidopsis thaliana* (Eckardt, 2013). A shortened form of the transporter, ZIFL1.3, is located exclusively in the plasma membrane of leaf stomatal guard cells and is involved in drought tolerance via stomatal closure, most likely by modulating potassium and proton fluxes in plant cells.

Another group of proteins affecting the plant response to abiotic stresses is the glycine‐rich RNA‐binding proteins (GRPs), of which heterogeneous ribonucleoprotein particle ‐like proteins are members. However, the functions of these proteins remain largely unknown. In *Arabidopsis*, GRP7 negatively affects plant development in response to drought stress during seed germination, seedling growth, and stomatal movement (Kim et al., 2008). GRP7, as well as GRP8, binds to mRNA, which affects splicing and microRNA biogenesis (Koster et al., 2014; Streitner et al., 2008).

The *Arabidopsis* splicing factor protein STABILIZED1, a homolog to the human U5 small nuclear ribonuclearproteins ‐associated protein, is involved in pre‐mRNA splicing and turnover of unstable transcripts. It also affects microRNA biogenesis via pri‐miRNA splicing. This protein is a regulator of plant responses to drought as well as to other abiotic stresses (Ben Chaabane et al., 2013; Kim et al., 2017; Lee et al., 2006; Shin et al., 2011).

The *CAP‐BINDING PROTEIN 20* (*CBP20*) and *CAP‐BINDING PROTEIN 80*/*ABSCISIC ACID HYPERSENSITIVE1* (*CBP80*/*ABH1*) genes were found to affect plant sensitivity to ABA and the ABA signaling pathway (Hugouvieux et al., 2001, 2002; Pieczynski et al., 2013). CBP20 and CBP80 form a nuclear cap‐binding complex that binds to the cap structure and affects pre‐mRNA splicing, mRNA stability, and microRNA biogenesis (Flaherty et al., 1997; Izaurralde et al., 1994; Kmieciak & Jarmolowski, 2002; Lewis et al., 1996; Pieczynski et al., 2013; Raczynska et al., 2010; Szarzynska et al., 2009). *Arabidopsis cbp20* and *cbp80*/*abh1* mutants were found to be hypersensitive to ABA during germination (Hugouvieux et al., 2001; Reyes & Chua, 2007), moreover, adult *cbp20* and *cbp80*/*abh1* mutants exhibit drought tolerance (Pieczynski et al., 2013). The responses concerning *CBP80*/*ABH1* and *CBP20* gene activity and drought tolerance are evolutionarily conserved. In crop species, inactivation of the *CBP80*/*ABH1* gene in potato and the *CBP20* gene in barley results in drought‐tolerant phenotypes (Daszkowska‐Golec et al., 2013; Pieczynski et al., 2013). A model explaining the role of the plant *CBP80/ABH1* gene in drought tolerance has been proposed. In this model, the lack of CBP80/ABH1 protein downregulated the level of miR159, which targets the mRNA of the TFs MYB33 and MYB101. MYB TFs have already been reported to function in ABA signaling and plant responses to drought (Abe et al., 1997; Casaretto et al., 2016; Jung et al., 2008; Lee et al., 2006; Reyes & Chua, 2007). As a result of miR159 downregulation in *cbp80*/*abh1* mutant plants, increased levels of MYB33 and MYB101 occurred, which probably resulted in increases in MYB33 and MYB101 TFs. This effect has been proposed as crucial for plant tolerance to drought (Pieczynski et al., 2013).

This study aimed to confirm the above‐described model showing that the level of selected MYB TFs affects plant tolerance to drought. We show that downregulation of *MYB33*, *MYB65* (as closely related to MYB33 in the sequence and with assumed redundancy in functionality), and *MYB101* gene expression renders *Arabidopsis* plants more sensitive to drought. Consequently, overexpression (OE) of these genes in *Arabidopsis* and potato plants resulted in an increase in their drought tolerance. Plants with downregulated *MYB33*, *MYB65*, and *MYB101* expressions were hyposensitive to ABA and closed their stomata in response to higher ABA concentrations than wild‐type (WT) plants. In contrast, plants overexpressing *MYB33*, *MYB65*, or *MYB101* are hypersensitive to ABA and close their stomata in response to lower ABA concentrations than WT plants. Thus, the lack of CBP80/ABH1 impairs miR159 biogenesis, resulting in an increase in the studied MYB TFs, which results in improvements to plant responses to drought.

## 2 MATERIALS AND METHODS

### 2.1 Experimental design

To reveal the role of MYB33, MYB65, and MYB101 in *Arabidopsis* and potato, transgenic plants over‐expressing each of these TFs have been obtained. Transgenic plants with downregulation of MYB33, MYB101, and knock‐down of MYB65 in the case of *Arabidopsis* were selected from appropriate SALK mutant accessions. Obtained and characterized plants were subjected to drought experiments. Relative water content (RWC), stomata aperture, and cuticle thickness in all transgenic plants were measured.

### 2.2 Plant material and growth conditions

*Arabidopsis thaliana* ecotype Columbia‐0 WT plants were used as controls and for transformation with constructs for genetic OE. WT plants; insertion mutants from the SALK Institute collection (accession numbers: SALK_053624, SALK_042186, SALK_058312, and SALK_015891, which have a downregulated *MYB33* gene; SALK_063552, SALK_112364, and SALK_081162, which have a downregulated *MYB65* gene; and SALK_061355, which has a downregulated *MYB101* gene); and *MYB33*, *MYB65*, and *MYB101* OE transgenic *Arabidopsis* plants were grown in soil (Jiffy‐7 42 mm; Jiffy Products International AS, Stange, Norway) in growth chambers (Sanyo/Panasonic, Japan) that had a 16‐h day length (150–200 μmol/m^2^/s), a constant temperature of 22°C and 70% humidity. All experiments, except those involving the selection of transformants, were conducted on 4‐week‐old plants and with three biological replicates (plants were sown at three separate times). After they were sterilized in 10% sodium hypochlorite in 70% EtOH solution, the seeds from transformed *Arabidopsis* plants were grown on half‐strength Murashige‐ Skoog (MS) media with 0.8% agar square plates, to which hygromycin B was added as a selective marker. After selection, the seedlings were transferred to Jiffy pots and grown to seeds, which were collected (Szarzynska et al., 2009).

*S. tuberosum* cultivar Désirée plants were used in this study. The plants were grown and propagated in vitro as described by Strzelczyk‐Zyta (2017) in glass tubes (ø25 mm, height 150–200 mm) filled to approximately one fifth of the height with half‐strength MS medium with 1% agar, with cellulose corks secured with Parafilm™, in a constant temperature of 21 ± 1°C, with 24 h light (150–200 μmol/m^2^/s^1^). The *Agrobacterium*‐mediated transformation of potato plants was carried out as described in Pieczynski et al. (2013). Potato plants were transferred from in vitro cultures into cuvettes (35 × 50 × 12 cm) filled with soil. To mitigate stress derived from transplanting the plants from in vitro culture to greenhouse conditions, plants were kept under a plastic cap for about 2 weeks. After which, the caps were removed, and the plants were grown for another 4 weeks. Plants were then transferred into 15 cm diameter pots filled with soil and grown in a greenhouse until they were 30–35 cm tall. For each line, six plants of equal size were transferred to a growing chamber (16/8 h, 23°C/15°C day/night; light intensity above the canopy 120 μmol/m^2^/s^1^).

### 2.3 Nucleic acid isolation, cDNA synthesis and PCR

Total RNA from 3‐week‐old or 4‐week‐old plant leaves was isolated from *Arabidopsis* plants and in vitro potato cultures using a Direct‐zol RNA MiniPrep Kit (Zymo Research). A TRIzol™ reagent (Invitrogen)‐based protocol was used for the reverse transcription reaction performed with Superscript™ III Reverse Transcriptase (Invitrogen), and oligo‐dT was used as a primer (Szarzynska et al., 2009) in accordance with the protocol provided by the manufacturer. The RNA was then cleaned with Turbo™ DNase (Invitrogen) according to the provided protocol.

Bands containing polymerase chain reaction (PCR) products were cut from the gel, and/or PCR products after the reaction were extracted from the gel and cleaned with a GeneJET Gel Extraction and DNA Cleanup Kit (ThermoFisher Scientific) according to the protocol provided by the manufacturer. The products were subsequently cloned into a pGEM®‐T Easy vector (Promega). Real‐time PCR and quantative PCR (qPCR) calculations were performed as previously described by Szarzynska et al. (2009) and Sierocka et al. (2011). The Mann–Whitney *U*‐test was used for statistical analyses. The following *p‐*values were set as statistically significant: *p* < 0.05; *p* < 0.01; *p* < 0.001.

Genomic DNA was isolated from *Arabidopsis* plants for genotyping with a “fast” protocol by grinding small leaves in a 1.5 ml Eppendorf tube via a small plastic pestle and extraction buffer containing 10% sodium dodecyl sulfate (SDS), EDTA, NaCl, and Tris–HCl, after which the DNA was precipitated with isopropanol. According to the provided protocol, plasmid DNA was isolated with a GenElute™ Plasmid Miniprep Kit (Sigma Aldrich).

### 2.4 Genetic constructs

Constructs for OE were prepared with a pMDC32 Gateway™ binary plasmid containing the CaMV 35S promoter, the hygromycin B phosphotransferase gene as a selective gene, and kanamycin and rifampicin resistance genes (Curtis & Grossniklaus, 2003). The cDNA sequences of the genes of interest were inserted into pENTR™/D‐TOPO™ (Invitrogen) plasmids and transferred to pMDC32 using the Gateway™ LR Clonase™ II (Invitrogen) technique. The nucleotide sequences of *A. thaliana MYB33* and *MYB65* with mutated miR159 recognition site genes were kindly provided by Prof. A. A. Millar (Millar & Gubler, 2005) and were amplified using the primers kMYB33 a.thATG_F/R and kMYB65 a.th F/R (Table 1). The *MYB101* gene from *Arabidopsis* (AT2G32460) and both the *MYB33* (PGSC0003DMT400058426) and *MYB65* (PGSC0003DMT400015156) gene sequences (the accession numbers refer to the SPUD database) from *S. tuberosum* were amplified from cDNAs using primers designed via Primer Blast (https://www.ncbi.nlm.nih.gov/tools/primer-blast/; kMYB101a.thATG_F/R; kMYB65s.tATG_F/R; kMYB33s.tATG_F/R). The miR159 recognition site in these genes was altered (keeping the unaltered amino acid sequences) by the Millar group, and introduced by directed mutagenesis using a QuikChange II Site‐Directed Mutagenesis Kit (Agilent Technologies); the primers m33F/R, m65F/R, and m101F/R were used (Table 1). To introduce gene sequences into the pENTR/D‐TOPO™ plasmid restriction enzyme sites for NotI and AscI, the primers kMYB33a.thRE_ATG_FLAG_F/A33_ASCI, kMYB65a.thRE_ATG_FLAG_F/A65_ASCI, kMYB101a.thRE_ATG_FLAG_F/A101_ASCI, kMYB33s.tRE_ATG_FLAG_F/S33_ASCI, and kMYB65s.tRE_ATG_FLAG_F/S65_ASCI were used (Table 1). In addition, a FLAG tag was also introduced into the 5′ sites of the given MYB cDNA coding sequence. The *Agrobacterium tumefaciens* AGL1 strain was used for the floral dip transformation of *A. thaliana* (Clough & Bent, 1998), and the LBA4404:rif^R^ pAL4404 strain was used for *S. tuberosum* transformation (Millam, 2006). The potato transformation procedure was performed as described by Wyrzykowska et al. (2016).

**TABLE 1:** Oligonucleotides

Name	Sequence (5'→3')	Product
kMYB101 s.tATG_F	ATGGCCCCCGATGATAGAGGAATGAG	MYB101 CDS from *S. tuberosum*
kMYB101 s.tATG_R	CTAACAAAAAGGAGGCATATTGTTCC	
kMYB101 a.thATG_F	ATGGATGGTGGTGGAGAGACGACG	MYB 101 CDS from *A. thaliana*
kMYB101 a.thATG_R	CTAACAGATGCTAGGCATGTTGCTCCA	
kMYB101 a.thRE_ATG_FLAG_ F	ATAAGAATGCGGCCGCATGGACTACAAAGACGATGACGACAAGGATGGTGGTGGAGAGACGACG	Insertion of restriction enzymes sites and FLAG tag to *Arabidopsis* MYB101 construct
A101_ASCI	ACAGATGCTAGGCATGTTGCTCCAATAGTCAGGGCGCGCCAAA	
kMYB65 s.tATG_F	ATGACAAGTGAAAGCGATGACAG	MYB 65 CDS from *S. tuberosum*
kMYB65 s.tATG_R	TCAGTACCAGATGGATCTTACAG	
kMYB65 s. tRE_ATG_FLAG_ F	ATAAGAATGCGGCCGCATGGACTACAAAGACGATGACGACA AGACAAGTGAAAGCGATGACAGGATGAC	Insertion of restriction enzymes sites and FLAG tag to potato MYB65 construct
S65_ASCI	GTACCAGATGGATCTTACAGTCAGGGCGCGCCAAA	
kMYB65 a.th F	ATGAGTTACACGACGGCGACTGCT	MYB 65 CDS from *A. thaliana*
kMYB65 a.th R	TTACAGCGACCAAACAGGAGGC	
kMYB65 a.th RE_ATG_FLAG_ F	ATAAGAATGCGGCCGCATGGACTACAAAGACGATGACGA CAAGAGTTACACGACGGCGACTGCT	Insertion of restriction enzymes sites and FLAG tag to *Arabidopsis* MYB65 construct
A65_ASCI	GACCAAACAGGAGGCATATTGTTAGGGCGCGCCAAA	
kMYB33 s.tATG_F	ATGAGCATCACAAGTGAAACCG	MYB 33 CDS from *S. tuberosum*
kMYB33 s.tATG_R	CTACACGGCTGACATGGCATCCCA	
kMYB33 s. RE_ATG_FLAG_ F	ATAAGAATGCGGCCGCATGGACTACAAAGACGATGACGACAAGAGCATCACAAGTGAAACCGAGGAAAGG	Insertion of restriction enzymes sites and FLAG tag to potato MYB33 construct
S33_ASCI	CACGGCTGACATGGCATCCCTCAGGGCGCGCCAAA	
kMYB33 a.thATG_F	ATGAGTTACACGAGCACTGACAGTG	MYB 33 CDS from *A. thaliana*
kMYB33 a.thATG_R	TCAACAAACTATTTCAAGTGATGGTAAGG	
kMYB33 a.th RE_ATG_FLAG_ F	ATAAGAATGCGGCCGCATGGACTACAAAGACGATGACGACAAGAGTTACACGAGCACTGACAGTG	Insertion of restriction enzymes sites and FLAG tag to *Arabidopsis* MYB33 construct
A33_ASCI	AGGGCGCGCCAAA	
m33F	GAGCCCACATGGGCAATGAAGCTAGAATTGCCAAGCCTACAGAACCAGACAGAAAACTGGGGCTC	Mutagenesis of MYB33 gene
m33R	GAGCCCCAGTTTTCTGTCTGGTTCTGTAGGCTTGGCAATTCTAGCTTCATTGCCCATGTGGGCTC	
m65F	GAGCCCTCATGGGCAAAGAAGCTAGAATTGCCAAGCCTACAGAGTCCGATTGCAAGTTGGGGCTT	Mutagenesis of MYB65 gene
m65R	AAGCCCCAACTTGCAATCGGACTCTGTAGGCTTGGCAATTCTAGCTTCTTTGCCCATGAGGGCTC	
S33_cDNA_wewF1	TCTGGCCCAGGGTCATAGAG	Sequencing constructs containing MYB33 from *S. tuberosum*
S33_cDNA_wewR1	TTAAATAGGCATGGCCGCCT	
S65_cDNA_wewF1	CGTGCAGGCTTGCCAATTTA	Sequencing constructs containing MYB65 gene from *S. tuberosum*
S65_cDNA_wewR1	TACACCCAAGGATGAGGGGT	
A65_cDNA_wewF1	TGCCTTCCCCAAAGCAAATC	Sequencing constructs containing MYB65 gene from *A. thaliana*
A65_cDNA_wewR1	CGGAGTCGTCTTCCACTGAT	
Cyclophilin S.tub F	CTCTTCGCCGATACCACTCC	Cyclophilin gene from *S. tuberosum*
Cyclophilin S.tub R	TCACACGGTGGAAGGTTGAG	
3’ GAPDH F	TTGGTGACAACAGGTCAAGCA	3′ Fragment of Glyceraldehyde ‐3phosphate dehydrogenase (GAPDH) gene from *A. thaliana*
3’ GAPDH R	AAACTTGTCGCTCAATGCAATC	
5’ GAPDH F	TCTCGATCTCAATTTCGCAAAA	5′ Fragment of GAPDH gene from *A. thaliana*
5’ GAPDH R	CGAAACCGTTGATTCCGATTC	
Actin A.th F	GGTAACATTGTGCTCAGTGG	1 i3 exon from Actin2 gene from *A. thaliana*,
ActinA.th R	CTCGGCCTTGGAGATCCACA	
qMYB33ath_F3	AAGAATTCTCGTCGCCTGAA	Fragment of MYB33 from *A. thaliana* for qPCR
qMYB33ath_R3	ACAGGTGAATGTCGGTTTCC	
qMYB65ath_F	GCTTTTGCAGGGAATGTTGT	Fragment of MYB65 from *A. thaliana* for qPCR
qMYB65ath_R	GCATCACTCATGCTGCTTGT	
qMYB101ath_F	TGGAACACGAGGCTAAAGAGA	Fragment of MYB101 from *A. thaliana* for qPCR
qMYB101ath_R	AAGTTGCTCTTTGGGGATGAT	
qMYB33Stub_F	GGACTTGCTCGTTGTGGTAAA	Fragment of MYB33 from *S. tuberosum* for qPCR
qMYB33Stub_R	AACTATCCGCTGCTCTTCCTC	
qMYB65Stub_F	TTCTTTTCCAACCTGCCATC	Fragment of MYB65 from *S. tuberosum* for qPCR
qMYB65Stub_R	CCATGAGGGCTCTGAAGAAG	
SALK_015891 LP	CCAATGCAAGAAGAAGTTTGC	Genotyping of insertion mutant MYB33
SALK_015891 RP	CATCGCAGGTAGAGGAGTCAG	
SALK_081162 LP	AAAAATAATTATGGATAAAGGTTTGG	Genotyping of insertion mutant MYB65
SALK_081162 RP	ATTCCCTCCATCCCTTTCTTC	
SALK_061355 LP	CCATATCATTTGCCATAACCG	Genotyping of insertion mutant MYB101
SALK_061355 RP	TAGTAGGATCCACATGTGCCC	
LBb1.3	ATTTTGCCGATTTCGGAAC	Genotyping of insertion mutants

### 2.5 RWC measurements

The RWC was measured as described previously (Pieczynski et al., 2013). All measurements for *Arabidopsis* were obtained as the average of five leaves of similar size from the middle part of the rosette and taken from two or three individual plants. The RWC was measured during six consecutive days of the drought experiment after the cessation of watering. For potato plants, the RWC measurements were obtained as the average of four leaves (third and fourth complex leaf) that were similar in size and taken from six individual plants on Day 0 and Day 6 of drought. Statistical analysis was carried out using Mann–Whitney tests.

### 2.6 Analysis of stomatal density

Experiments were carried out using fully developed rosette leaves of *Arabidopsis* plants. Cleared epidermal fragments peeled from *Arabidopsis* abaxial leaf surfaces were prepared according to the methods of Pei et al. (1997, 2000) and as described previously by Pieczynski et al. (2013) and Pei et al. (1997). The adaxial side was imprinted in clear nail polish. Images of the specimens on microscope slides were taken with a Nikon Eclipse Ti light microscope and a DS‐Fi1c‐U2 camera, a Plan Fluor 10x DIC L N1 optical system, or a Zeiss AXIO Observer Z1 system. Counts were made using at least 10 images from 3 to 5 individual plants and averaged per square millimeter surface using NIS‐Elements Advanced Research software (Nikon Instruments Europe B.V.). The number of stomata was counted on fully expanded apical leaflets from 5‐week‐old potato plants cultivated in the greenhouse. Three leaflets were detached from each of the three plants, and adaxial and abaxial epidermal peels were removed and examined under a Nikon Eclipse E200 binocular microscope. Stomatal density was measured within an area of 1 mm^−2^ in three separate locations on each leaflet. Statistical analysis was carried out using the Mann–Whitney test.

### 2.7 Determination of trichome density

The adaxial surfaces of *A. thaliana* leaves were examined using a binocular microscope (Leica Microsystems M60 equipped with an MC170 HD camera). Trichomes were counted using at least 10 images of *Arabidopsis* leaves from 3 to 5 plants and averaged per square millimeter surface using NIS‐Elements Advanced Research software. The adaxial and abaxial leaflet surfaces of potato plants were evaluated for trichome density under a Nikon Eclipse E200 binocular microscope. The trichomes were counted on three 5‐week‐old potato plants cultivated in the greenhouse. Three leaflets were taken from each plant, and the trichome density was measured within 1 mm^−2^ in three separate locations on each leaflet. Statistical analysis was carried out using the Mann–Whitney test.

### 2.8 Stomatal movement analysis

Stomatal aperture measurements were measured as described previously (Hugouvieux et al., 2001; Pei et al., 2000; Pieczynski et al., 2013; Savvides et al., 2012). Images of the epidermal peels and measurements of stomatal aperture were analyzed similarly to those used for stomatal density (see above). For all ABA concentrations, at least 10 images of *Arabidopsis* leaves from 2 to 3 plants and average measurements were assessed using the NIS‐Elements Advance Research program (Zhang et al., 2016). Statistical analysis was carried out using the Mann–Whitney test.

### 2.9 Cuticle thickness analysis

The procedure for evaluating leaf cross‐sections and measuring cuticles via transmission electron microscopy was carried out according to Krzesłowska and Woźny (1996) and as previously described by Pieczynski et al. (2013). Statistical analysis was carried out using the Mann–Whitney test.

## 3 RESULTS

### 3.1 *Arabidopsis* plants with a *MYB33* , *MYB65*, or *MYB101* deficiency show a severe wilted phenotype upon drought and are hyposensitive to ABA

**FIGURE 1:**
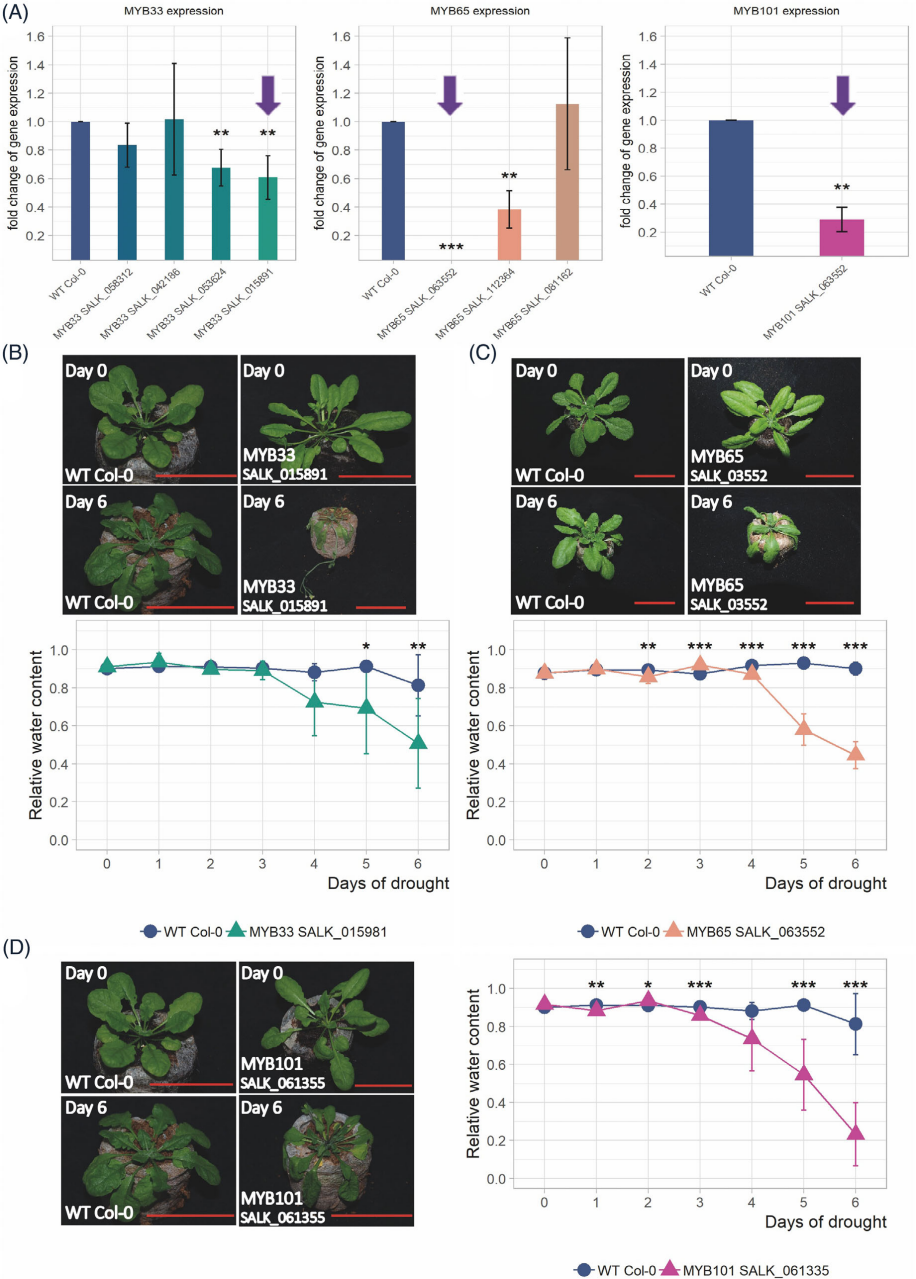
*Arabidopsis* plants with MYB33, MYB65, or MYB101 deficiency show a severe wilted phenotype during drought. (A) Levels of MYB33, MYB65, and MYB101 mRNA in selected mutant plants. The left graph shows the RT‐qPCR measurements of the downregulated MYB33 mRNA in four SALK lines, with line SALK_015891 exhibiting the most profound downregulation across all lines compared to that in the WT plants. The middle graph shows the level of MYB65 mRNA expression in three SALK lines, with the SALK_063552 line showing total knockdown of the MYB65 gene compared to that in the WT plants. The right graph shows MYB101 mRNA expression in the SALK_061355 mutant plant with 3.5 times lower expression compared with that in the WT plants. **p < 0.01; ***p < 0.001; Mann–Whitney *U* test. The labels of the select mutant lines are shown at the bottom of each graph. The blue arrows point to the select SALK mutants used further in this study. (B–D) After 6 days of drought, the *myb33*, *myb65*, and *myb101* mutant plants showed a significant wilted phenotype (upper panels) in comparison to the WT plants. RWC experiments (lower panels) show substantially high water loss in all three *MYB* mutant plants during the course of drought, starting on the 3rd day after the cessation of watering. The images show plants on Days 0 and 6 of the drought experiments involving both WT and mutants in the upper panels of B, C, and in the left panel of D. The graphs show the RWC results during the time course of drought, measured each consecutive day (blue line: WT plants; green line: *myb33* mutant plants [B], salmon line: *myb65* mutant plants [C], magenta line: *myb101* mutant plants [D]). The data are shown as the means ± sds of *n* = 3 independent experiments; Mann–Whitney test, *p* value: *p < 005; **p < 0.01; ***p < 0.001. Scale bar: 50 mm. RWC, relative water content; WT, wild‐type.

To identify *Arabidopsis* mutants with knocked out or knocked down expression of the investigated *MYB* genes, homozygous plants were obtained from seeds from the SALK collection (including four mutant lines with different T‐DNA insertion sites for the *MYB33* gene, three lines for the *MYB65* gene and one for the *MYB101* gene, Figure S1). The line with the lowest expression level of the *MYB33* gene (SALK_015891), the line with no expression of the *MYB65* gene (SALK_063552), and the line showing strong downregulation of *MYB101* expression (SALK_061355) were selected for further analyses (Figure 1A).

Four‐week‐old homozygous plants were subjected to drought stress by complete water cessation for 6 days, after which the RWC of the plant leaves was measured. Compared to WT plants with downregulated or knocked out *MYB33*, *MYB 101*, or *MYB65* genes exhibited a significant decrease in their tolerance to water deficit. This observation was strengthened by RWC measurements that show approximately 2‐fold lower levels of water content in all mutant lines compared to WT plants (Figure 1B–D).

**FIGURE 2:**
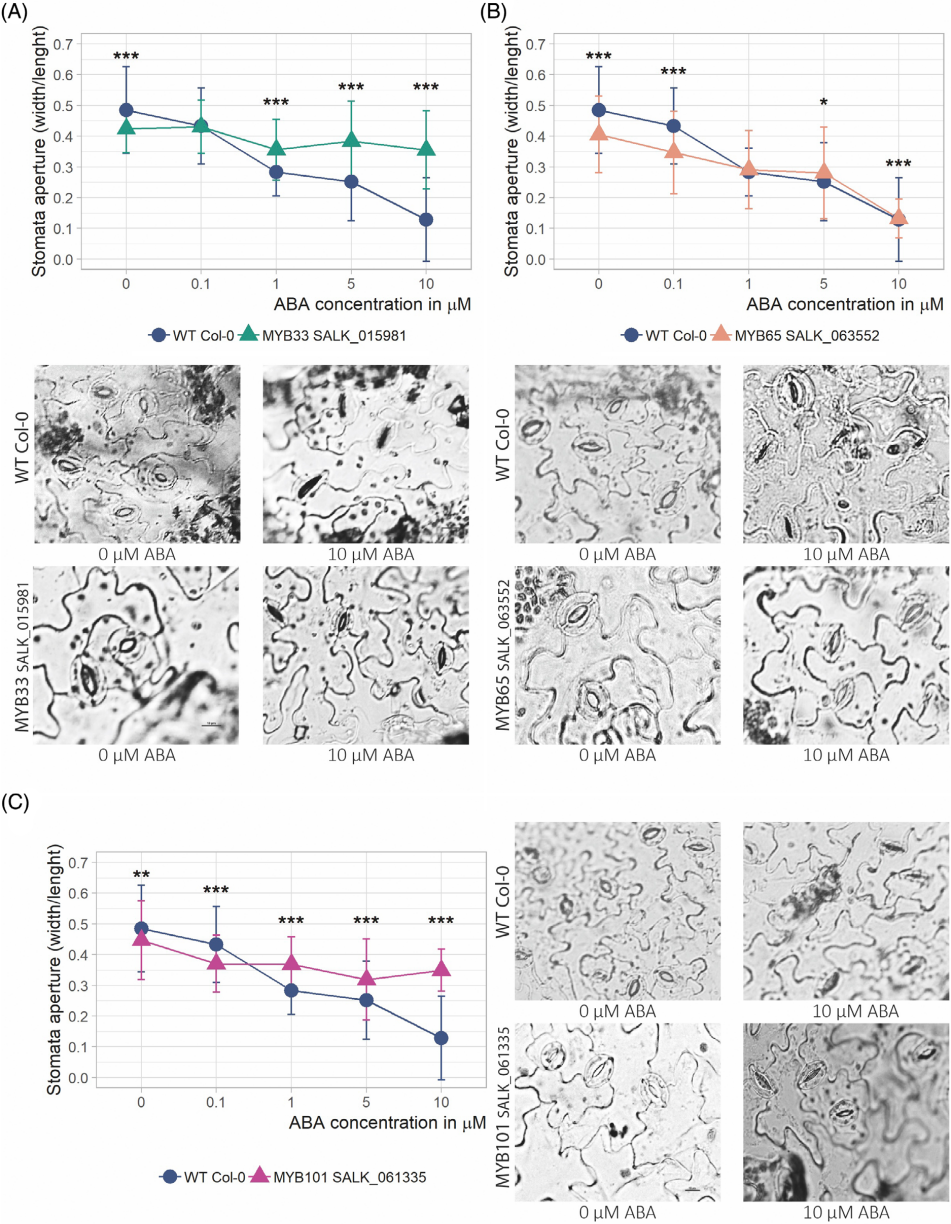
*Arabidopsis* mutant plants exhibiting MYB33, MYB65, or MYB101 deficiency show an ABA‐hyposensitivity phenotype. (A) Stomatal closure in ABA‐hyposensitive mutant plants with MYB33 deficiency compared to WT plants. The WT and mutant plants were treated with increasing concentrations of ABA (upper panel: the graph shows the stomatal aperture). WT: blue line; *myb33* mutant: green line. (B, C) Stomatal closure in ABA‐hyposensitive mutant plants with a MYB65 (B) and MYB101 (C) deficiency compared to WT plants. WT: blue line; *myb65*: salmon line; *myb101*: magenta. The description of the upper and lower panels is the same as that in the case of the *myb33* mutant. (A–C) The lower panels show light micrographs of the stomata in the WT and *myb33*, *myb65*, and *myb101* mutant plants treated with 0 and 10 μM ABA. The data are shown as the means ± sds of *n* = 3 independent experiments, with 30 stomata per data point. Scale bar: 10 μm; Mann–Whitney test, *p* value: *p < 0.05; **p < 0.01; ***p < 0.001. ABA, abscisic acid; WT, wild‐type.

These results reinforced the hypothesis that the levels of the *MYB33, MYB65*, and *MYB101* genes are involved in the plant response to drought. To uncover the reasons behind the strong wilting of the mutant plants with the downregulation of the studied MYB TFs, we investigated selected morphological and physiological traits that often reflect plant strategies for coping with water deficiency stress and shown in the case *of cbp80/abh1 Arabidopsis* and potato mutants, which were strongly affected. These traits include stomatal (on both sides of the leaf blades) and trichome density, cuticle thickness and stomatal response to ABA. In our previous studies, a lack of *CBP80*/*ABH1* gene expression led to increased drought tolerance, downregulation of stomatal density on the upper leaf blades, increased numbers of stomata on the bottom leaf blades, increased numbers of trichomes on the upper leaf blades, increased cuticle density, and hypersensitivity to ABA‐induced stomatal closure (Pieczynski et al., 2013). In the case of the *myb33*, *myb65*, and *myb101 Arabidopsis* mutant plants, we expected either no effects or the opposite effects as those observed for the *cbp80*/*abh1* mutants in our previous studies. There were no clear effects on stomatal or trichome numbers or cuticle density (Figures S2 and S3). However, the fully opened stomatal aperture was smaller in the transgenic plants than in the WT plants (13% in the case of the *myb33* mutant plants, 20% in the case of the *myb65* mutant plants, and 7.8% in the case of the *myb101* mutant plants). Moreover, the stomata of the mutant plants with downregulated expression of the *MYB33, MYB65*, and *MYB101* genes remained open even under 10 μM ABA concentrations, while the WT plants started closing their stomata in response to concentrations as little as 0.1 μM ABA until they were completely closed in response to 10 μM ABA (Figure 2). Thus, the *myb33*, *myb65*, and *myb101 Arabidopsis* mutant plants exhibited hyposensitivity to ABA with respect to stomatal closure, which is in agreement with our expectations concerning the action of *CBP80*/*ABH1* upstream of the regulatory pathway controlling ABA‐dependent stomatal closure.

### 3.2 *Arabidopsis thaliana* transgenic plants overexpressing the *MYB33* , *MYB65* , and *MYB101* genes from *Arabidopsis* and the *MYB33* gene from *S. tuberosum* are tolerant to drought and hypersensitive to ABA

To determine whether *Arabidopsis* plants overexpress the *AtMYB33*, *AtMYB65*, *and AtMYB101* genes exhibit drought‐tolerant phenotypes, we cloned the appropriate cDNAs into a pMDC32 binary plasmid containing both the CaMV 35S promoter and the hygromycin B phosphotransferase gene as a selective marker. Additionally, a FLAG tag was added to the 5′ end of each coding sequence. The same constructs were prepared with *MYB33* and *MYB65* cDNAs from potato (*StMYB33* and *StMYB65*). To our knowledge, there is no *MYB101* gene in the potato genome. To avoid post‐transcriptional silencing of the introduced MYB transcripts by microRNA 159, the microRNA 159 recognition site of the overexpressed *Arabidopsis* and potato cDNAs was mutated at the nucleotide level, ensuring that the amino acid sequences were unaffected (Millar & Gubler, 2005) (Figure S4A). The constructs were subsequently introduced into *Arabidopsis* WT plants, and homozygous plants were selected. In the case of the *AtMYB101 Arabidopsis* transgenic plants, we were unable to obtain homozygous plants. We, therefore, analyzed the siliques of the *AtMYB101* OE transformants. The transformants showed empty spaces where seeds should develop and/or they contained underdeveloped seeds compared to WT siliques, which contained a septum fully stacked with properly developed green seeds (Figure S5). These results indicate that overly high *AtMYB101* gene OE negatively affects fertilization and induces embryo lethality. We decided to continue our studies on the role of *AtMYB101* in plant drought tolerance using heterozygous plants, which were genotyped before each experiment. We also did not obtain *Arabidopsis* plants overexpressing potato *MYB65*. The expression of transgenic cassettes in selected *Arabidopsis* transgenic lines was tested using RT‐PCR and western blotting (Figure S4B,C). For subsequent experiments, we selected three transgenic lines with the highest expression of each introduced transgene (A33 2‐1, A33 6‐4, A33 6‐6, A65 3‐1, A65 3‐2, A65 5‐4, A101 2‐2‐2, A101 2‐11‐2, A101 2‐11‐4, S33‐1, S33‐5, S33 3‐11).

**FIGURE 3:**
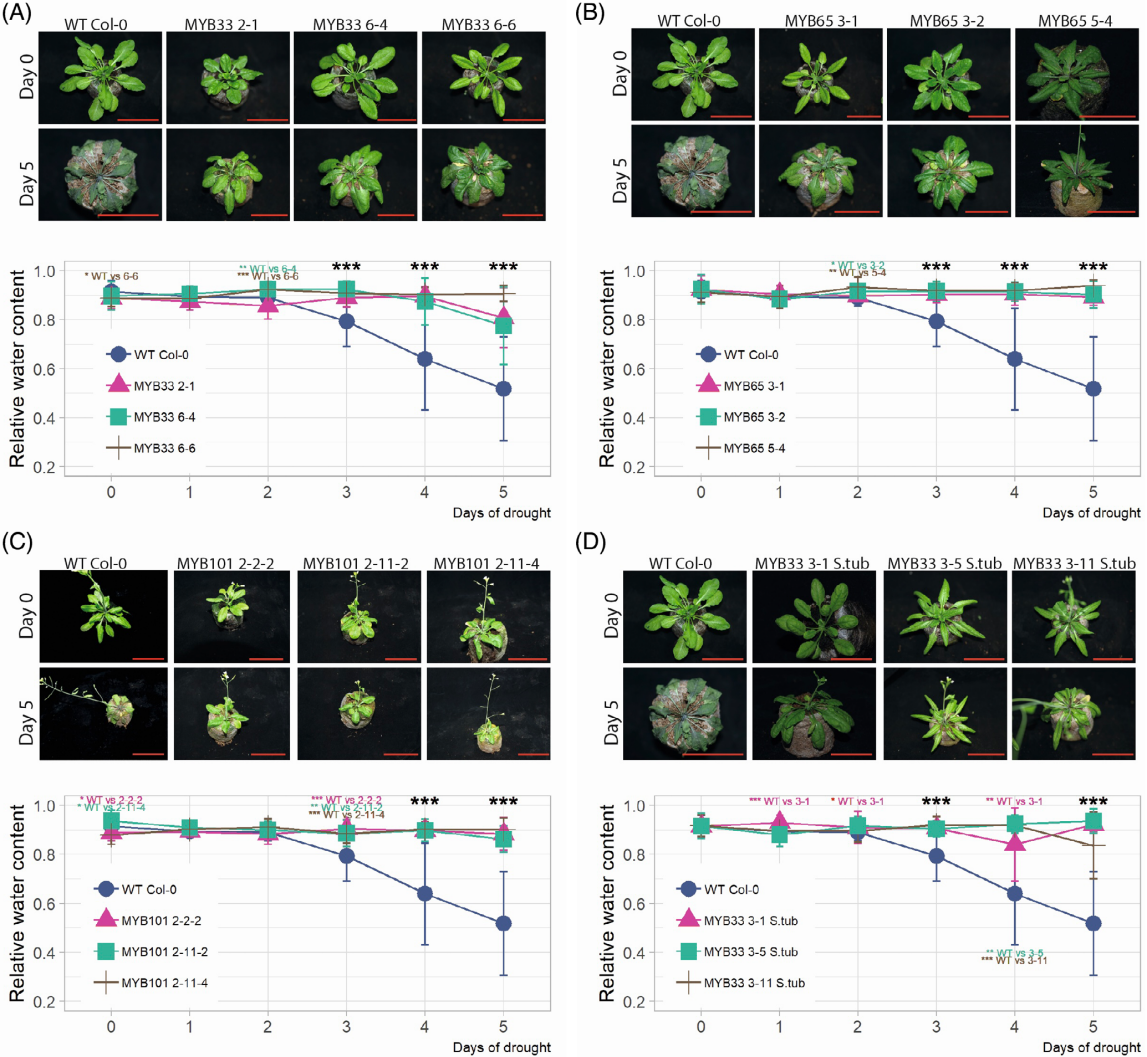
*Arabidopsis* transgenic plants overexpressing the *MYB33*, *MYB65*, or *MYB101* gene from *Arabidopsis* and the *MYB33* gene from *S. tuberosum* are resistant to drought. (A) The upper panel shows plants from three independent transgenic *Arabidopsis* lines overexpressing the *MYB33* gene (MYB33 OE) compared to WT plants. The plants are shown on Day 0 (upper row) and Day 5 (lower row) after water cessation. The lower panel graph shows the RWC during the time course of drought, measured each consecutive day (blue line: WT plants; other colored lines: plants from select independent transgenic lines overexpressing the *MYB33* gene). (B, C) The same experiments were performed for the *MYB65* and *MYB101* OE mutants. (D) Experiments performed for the mutants overexpressing *Solanum tuberosum MYB33* gene. Experiments of A, B, and D were performed in parallel using the same WT plant control. (A–D) The RWC data are shown as the means ± sds of *n* = 3 independent experiments; Mann–Whitney test, *p* value: *p < 0.05; **p < 0.01; ***p < 0.001. Scale bar: 50 mm. OE, overexpression; RWC, relative water content; WT, wild‐type.

**FIGURE 4:**
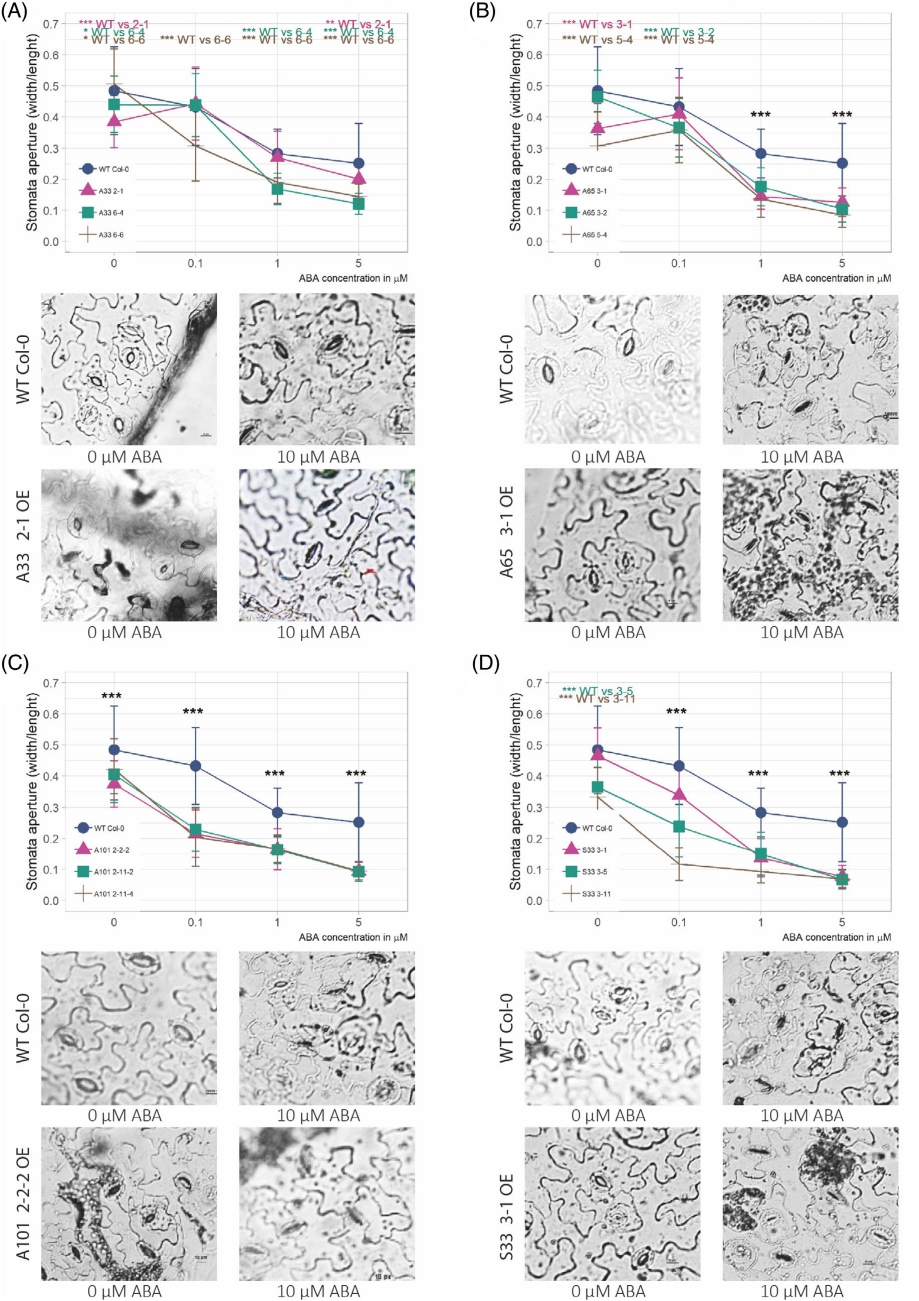
*Arabidopsis* mutants overexpressing the *MYB33*, *MYB65*, or *MYB101* gene from *Arabidopsis* and the *MYB33* gene from *Solanum tuberosum* show an ABA‐hypersensitive phenotype. (A) Stomatal closure in ABA‐hypersensitive mutant plants with *MYB33* OE compared to WT plants. The WT and mutant plants were treated with increasing concentrations of ABA (upper panel: the graph shows the stomatal aperture). WT: blue line; other colors: select transgenic mutant plants overexpressing *MYB33*. The lower panel shows light micrographs of the stomata in the WT plants and *MYB33* OE mutants treated with 0 and 5 μM ABA, respectively. (B, C, D) Stomatal closure in ABA‐hypersensitive mutant plants with *MYB65* (B), *MYB101* (C), and *S. tuberosum MYB33* (D) overexpression compared to WT plants. WT: blue line; other colors: select transgenic mutant plants overexpressing *MYB65* (B) or *MYB101* (C) or *S. tuberosum MYB33* (D). The description of the upper and lower panels is the same as that in the case of the *MYB33* OE mutant. (A–D) The stomatal closure measurement data are shown as the means± sds of *n* = 3 independent experiments, with 30 stomata per data point; Mann–Whitney test, *p* value: *p < 0.05; **p < 0.01; ***p < 0.001. Scale bar: 10 μm. ABA, abscisic acid; OE, overexpression; WT, wild‐type.

Four‐week‐old *Arabidopsis* plants from the selected transgenic lines were subjected to drought stress by complete water cessation for 6 days, and the RWC of the leaves was measured. In each case, plants carrying the OE MYB construct showed significantly improved drought tolerance compared to WT plants. This observation is supported by RWC measurements showing higher water content levels in all mutant lines than in WT plants (Figure 3A–D). We examined the responsiveness of stomatal closure to ABA in all mutant plants and compared it with the WT plants. After a saturating humidity treatment, the stomatal apertures were smaller in all transgenic plants than in the WT plants whose stomata were fully opened (approximately 17%, 30%, 24%, and 27% for *AtMYB33* OE lines, *AtMYB65* OE lines, *AtMYB101* OE lines, and *StMYB33* OE lines, respectively). Furthermore, for all four genetic constructs, the plants exhibited significantly enhanced stomatal closure in response to ABA treatment compared to WT *Arabidopsis* plants (Figure 4A–D). Thus, OE of *AtMYB33*, *AtMYB65*, *AtMYB101*, and S*tMYB33* in *A. thaliana* plants resulted in hypersensitivity to ABA. These results are in full agreement with our previous studies on the role of *CBP80*/*ABH1* in the plant response to drought, emphasizing the upstream action of this protein in the ABA‐dependent signaling pathway, which leads to the regulation of *MYB33*, *MYB65*, and *MYB101* expression (Pieczynski et al., 2013).

Interestingly, the numbers of stomata on the adaxial and abaxial sides of leaf blades, as well as trichome density, were affected in all mutant plants overexpressing MYB TFs in the same way as that in the case of the *cbp80*/*abh1* mutants. When the abaxial leaf surfaces were inspected, the number of stomata on the *Arabidopsis* mutants showed a general increase compared to those on the WT plants, although this increase was not always statistically significant. The opposite trend, a statistically significant decrease in stomata number, was observed in the case of adaxial leaf surfaces in a majority of the mutant lines (Figure S6A). The number of trichomes on the adaxial surface of leaf blades also showed a statistically significant increase in the majority of mutant lines compared to WT plants (Figure S6B). In the case of the cuticle, we did not observe any morphological changes in its density; however, it was thinner in the majority of the mutant lines than in the WT plants (Figure S7). Thus, OE of the *MYB33*, *MYB65*, and *MYB101* genes suggests that stomatal and trichome density are at least partially under the control of the signaling pathway involving these genes and *CBP80*/*ABH1*, which acts upstream, while cuticle thickness is not.

### 3.3 *S. tuberosum* plants overexpressing MYB TFs show improved tolerance to drought

A previous study on the role of *CBP80*/*ABH1* in the plant response to drought revealed its evolutionary conservation between *Arabidopsis* and potato. To determine whether the role of select MYB TFs in the drought response is also conserved, we introduced the same genetic constructs containing the *MYB33*, *MYB65*, and *MYB101* genes from *A. thaliana* and *MYB33* and *MYB65* from *S. tuberosum* into plants of potato cultivar Désireé. After *Agrobacterium*‐mediated transformation via callus induction in vitro, selection of transformed tissue via cultivation on selective media, and regeneration of transgenic plants, we obtained 29 transgenic potato plants, which were transferred and cultivated in glass tubes: 12 *AtMYB33* OE plants, 6 *AtMYB65* OE plants, 4 *AtMYB101* OE plants, 6 *StMYB33* OE plants, and 1 *StMYB65* OE plant. The expression of each transgene was confirmed using RT‐PCR and the expression of the hygromycin B phosphotransferase gene as a control (Figure S8A,B). Three transgenic lines with the highest OE of each MYB TF studied were selected and transplanted into pots after adaptation to ex vitro conditions. Potato plants overexpressing *StMYB33* were smaller than WT potato plants. In the case of *StMYB65* OE plants, only one line was obtained. The *StMYB65* OE plants in vitro and those grown in pots showed a strong phenotype: they displayed a bushy shape, and extreme dwarfism, with shortened internodes in the stems and miniature leaves (Figure S9). Because of these morphological defects, it was decided not to include the *StMYB65* OE plants in subsequent studies.

**FIGURE 5:**
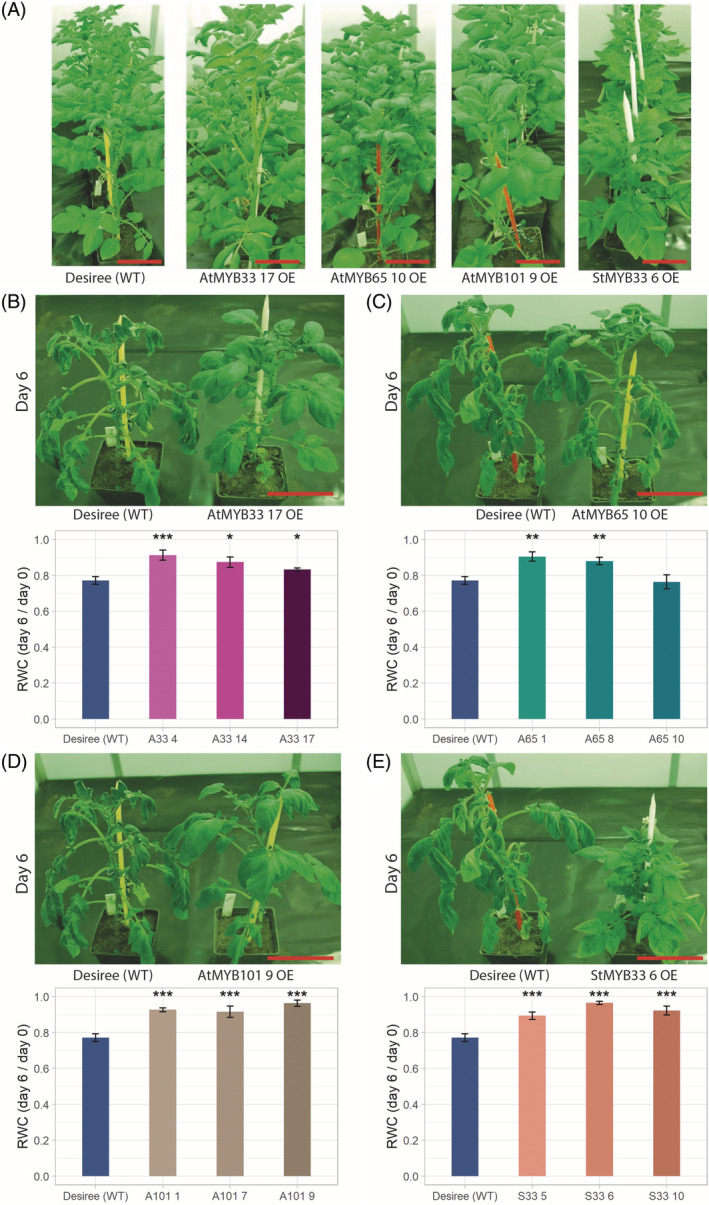
Transgenic lines of potato cultivar *S. tuberosum* cv. Désireé transgenic plants overexpressing the *MYB33*, *MYB65*, or *MYB101* gene from *Arabidopsis* and the *MYB33* gene from *S. tuberosum* are resistant to drought. (A) WT and mutant plants overexpressing *AtMYB33*, *AtMYB65*, *AtMYB101*, and *StMYB33* on Day 0 of drought. The labels shown at the bottom of each image represent the name of an individual transgenic line. (B) The upper panel shows transgenic potato plants overexpressing the *AtMYB33* gene (line AtMYB33‐17 OE) compared with WT plants. The plants are shown on Day 6 after water cessation. The lower panel shows the RWC measurements of leaves from WT and transgenic *AtMYB33* OE (A33) plants after drought stress. The RWC value of the control (Désireé: 0.77) is the value of the ratio between its water content on Day 6 and on the Day 0. Blue bars: WT plants; the other colored bars represent potato plants from independent transgenic lines overexpressing *AtMYB33*. (C–E) show the same data obtained for potato transgenic plants overexpressing *AtMYB65* (C), *AtMYB101* (D), and *StMYB33* (E), respectively. The figure descriptions are the same as the description in panel (A). (B–E) The RWC data are shown as the means ± standard error of the mean, *n* > 6 independent experiments; Mann–Whitney test, *p* value: *p < 0.05; **p < 0.01; ***p < 0.001. Scale bar: 15 cm. OE, overexpression; RWC, relative water content; WT, wild‐type.

**FIGURE 6:**
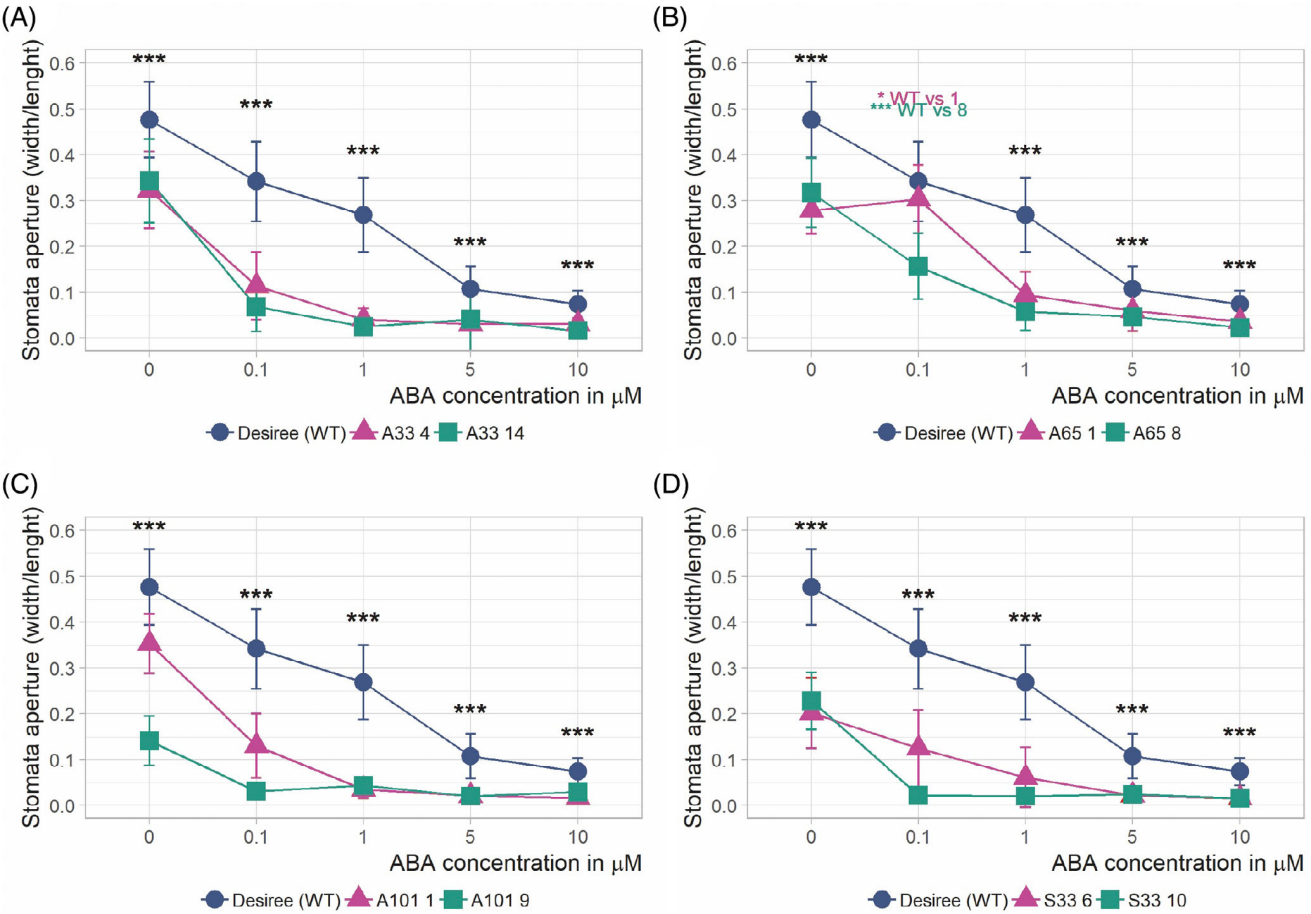
Transgenic lines of potato cultivar Désireé overexpressing the *AtMYB33*, *AtMYB65*, and *AtMYB101* genes and the *StMYB33* gene exhibit ABA hypersensitivity. (A) Stomatal closure in ABA‐hypersensitive transgenic potato plants with At*MYB33* OE compared to WT plants. The WT and mutant plants were treated with increasing concentrations of ABA. WT: blue line; other colors: select transgenic mutant lines overexpressing *AtMYB33*. (B–D) Graphs of the stomatal aperture of transgenic potato plants overexpressing *AtMYB65* (B), *AtMYB101* (C), and *StMYB33* (D). The figure descriptions are the same as the description in panel (A). The data are shown as the means± sds of *n* = 3 independent experiments, with 30 stomata per data point; Mann–Whitney test, *p* value: *p < 0.05; **p < 0.01; ***p < 0.001. Stomatal aperture was measured in two out of three independent transgenic lines for all OE constructs. ABA, abscisic acid; OE, overexpression; WT, wild‐type.

After the transgenic potato plants grew to 30–35 cm in height, they were subjected to drought stress by complete water cessation for 6 and 11 days, and the RWC of the plant leaves was measured. In each case, the plants carrying the OE MYB construct showed significant improvement in drought compared to the WT plants. This observation was supported by the RWC measurements, which showed higher levels of water content in all the mutant lines compared with the WT lines on the 6th day of drought (Figure 5A–E). However, plants experiencing 11 days of drought showed significantly increased water contents only in the *AtMYB101* and *StMYB33* lines (Figure S10). We also examined the responsiveness of stomatal closure to ABA in all potato mutant plants and compared it with that of the WT plants. The experiments were conducted in the same manner as in the case of *Arabidopsis* transgenic and WT plants. Two of three transgenic potato lines for each MYB TF subjected to drought were used for the ABA sensitivity experiment. After a saturating humidity treatment, stomatal apertures were smaller in all the transgenic potato plants than in the WT plants whose stomata were fully opened (approximately 30%, 37%, 47%, and 55% for plants overexpressing *AtMYB33*, A*tMYB65*, *AtMYB101*, and *StMYB33*, respectively), as occurred in the case of *Arabidopsis* transgenic plants overexpressing the studied MYB TFs. However, for all four genetic constructs, the potato plants exhibited significantly enhanced stomatal closure compared to WT potato plants (Figure 6A–D). Our experiments clearly show the evolutionary conservation of all MYB TFs studied in plants in response to drought.

Interestingly, the OE of the *MYB33*, *MYB65*, and *MYB101* genes in potato plants did not affect stomatal or trichome density in the same manner in all the transgenic lines, which is in contrast to that which occurred in the *Arabidopsis* plants overexpressing MYB TFs (Figure S11).

## 4 DISCUSSION

The results of this work support the model of the plant drought tolerance pathway that is induced by the lack of the *CBP80*/*ABH1* gene presented in our previous study (Pieczynski et al., 2013). As shown previously, the lack of this gene impairs miRNA159 induction, which is known to control *MYB33*, *MYB65*, and *MYB101* gene expression in *Arabidopsis* (Alonso‐Peral et al., 2010; Millar & Gubler, 2005; Reyes & Chua, 2007). The lack of miRNA159 induction resulted in elevated levels of *MYB33*/*MYB101* mRNAs, which purportedly increased plant tolerance to drought (Pieczynski et al., 2013). As expected, our recent results show that downregulation of *MYB33*/*65*/*101* expression resulted in decreased plant tolerance to drought, while OE of the same genes led to improved plant responses to water shortage. Moreover, the results of the present study show that plants with downregulated expression of *MYB33*/*65*/*101* genes were hyposensitive to ABA and closed their stomata at ABA concentrations greater than in WT plants. The opposite was observed in the case of plants overexpressing these MYB TFs. The role of MYB 33/101 TFs in ABA signaling has already been proven for seed germination in the case of *Arabidopsis* (Reyes & Chua, 2007). Recently in *Arabidopsis*, the role of mir159‐MYB33‐ABI5 in seed germination and drought response has been described (Guo et al., 2021; Jiang et al., 2022). However, the role of these TFs and MYB65 in the drought response has never been tested. Interestingly, the downregulation or upregulation of each individual MYB33/65/101 TF resulted in a strong response to drought, suggesting that these proteins are not redundant in their functions related to the response to water deficit. MYB33/65/101 belong to the same R2R3 subgroup of the MYB TF family. MYB33 and MYB65 proteins exhibit 58.4% identity in terms of their amino acid sequence; specifically, they share more than 90% identity in their R2R3 domains and 51% similarity in their carboxy‐terminal domains (Millar & Gubler, 2005). High similarity in a sequence may indeed suggest some redundancy in function. It was previously shown that MYB33 and MYB65 are involved in anther and pollen development (Dubos et al., 2010; Millar & Gubler, 2005). Moreover, it was suggested that both proteins act redundantly in these processes. However, MYB33 and MYB65 are not expressed equally in all plant tissues: MYB33 is expressed in all organs and tissues, with the highest expression of this gene occurring in germinating seeds, mature leaves, flowers, and carpels, while MYB65 is also expressed in all organs; however, the highest amount is detected only in germinating seeds and flowers. Generally, MYB33 is expressed at a higher level than MYB65, and MYB101 is expressed at the lowest level in all organs, with a slight increase in flowers and seeds during the first stages of germination (Winter et al., 2007). *Arabidopsis* MYB101, together with MYB97 and MYB120, has been shown to function as male factors that control pollen tube—synergid interactions during fertilization (Liang et al., 2013). Together, all these data show that MYBs 33/65/101 have different functions in various processes, possibly by controlling the expression of downstream genes. Moreover, the set of controlled genes may differ depending on the time and place of a given MYB TF action. These data suggest that although all three MYB TFs are expressed in whole plants, the level of their expression varies among the organs studied, which may affect different functions in plant development, strengthening the results obtained in this work. Moreover, these data show the lack of redundancy in their function in plants in response to drought.

The role of MYB TFs from the R2R3 subgroup in the drought response is not confined to the three MYB33/65/101 studied in this work. Previous studies on the *AtMYB44* gene (a member of the R2R3 subgroup) also revealed that this gene plays a role in the plant response to drought (Jung et al., 2008; Wu et al., 2019; Zhao et al., 2018). Plant overexpressing *MYB44* showed enhanced stomatal closure, which provided drought and salinity tolerance. Moreover, it was shown that *AtMYB44* OE resulted in reduced expression of genes encoding protein phosphatase 2C phosphatases that are known to be negative regulators of ABA signaling. Maize transgenic plants overexpressing *OsMYB55*, which also belongs to the R2R3 subgroup, also exhibited enhanced tolerance to water deficiency (Casaretto et al., 2016). OE of the potato MYB TF *StMYB1R‐1*, which belongs to another subgroup of MYB TFs (R1), also resulted in improved plant responses to drought and relatively rapid stomatal closure under drought (Shin et al., 2011). It was shown that StMYB1R‐1 enhanced the expression of genes involved in the regulation of water loss. It is highly probable that the mode of action of MYB33/65/101 in the *Arabidopsis* and potato responses to drought is involved in the same or other pathways that negatively affect ABA signaling and the regulation of water loss.

*Arabidopsis* MYB33, MYB65, and MYB101 were also shown to inhibit cell division in vegetative plant tissues and thus inhibit plant growth (Allen et al., 2007; Millar & Gubler, 2005). The most profound phenotypic effect was found in the case of a mutant carrying an inactivated *MIRNA159* gene. Our studies on transgenic potato plants harboring overexpressed potato MYB33 and MYB65 revealed a strong dwarf phenotype. Why we did not observe this phenotype in the case of potato and *Arabidopsis* plants overexpressing *Arabidopsis MYB33*, *MYB65*, or *MYB101* is not clear. The amount of individual overexpressed proteins for each transgenic line may play a crucial role here.

Drought limits the productivity of crop plants. Our results point to the selected MYB TFs being interesting, evolutionarily conserved targets for genetic manipulations aiming to obtain crop plants with improved plant tolerance to drought. In potato, tubers are sink organs, the most important agronomic potato trait. Damage to the photosynthetic apparatus and changes in photosynthetic processes due to drought result in decreasing tuber yield and quality. It will be important to show that the OE of the *MYB33*, *MYB65*, and *MYB101* genes is a potential way to increase potato yields under drought. Identifying target genes acting downstream of these MYB genes will also be important. Such an approach will allow the selection of genes with a narrower range of functions than MYB TFs have and the avoidance of undesired “off traits” in crop plants.

## AUTHOR CONTRIBUTIONS

Anna Wyrzykowska carried out the majority of experiments including mutant plant construction and drought experiments. Dawid Bielewicz took part in the discussion, prepared figures, and calculated statistics. Patrycja Plewka took part in the ABA ‐dependent stomata closure experiments and in the manuscript preparation. Dorota Sołtys‐Kalina participated in evaluation of RWC tests in the case of potato plants. Iwona Wasilewicz‐Flis participated in receiving of potato plant material. Waldemar Marczewski participated in discussions and manuscript preparation. Artur Jarmolowski took part in the experiment design and manuscript preparation. Zofia Szweykowska‐Kulinska created the concept of the work, designed experiments, and wrote the manuscript.

## CONFLICT OF INTEREST

The authors declare no conflict of interest.

## Supporting information


**FIGURE S1** *Arabidopsis MYB33*, *MYB65*, and *MYB101* gene T‐DNA insertion lines for analysis of gene knock‐down or downregulation.
Schematic graphs represent *MYB33*, *MYB65*, and *MYB101* gene structures. Gray color represents gene promotor and 3′ flanking region, red color represents 5′ and 3’ UTRs, orange color represents exons, and blue color represents intron. SALK numbers are given at the left side of each gene harboring T‐DNA insertion. Localization of T‐DNA insertion is marked in the each gene structure. Violet arrows point T‐DNA insertion lines for further analysis.

**FIGURE S2** Stomata and trichomes density on the surfaces of *Arabidopsis* leaves are differentially affected in the *Arabidopsis* mutant plants with the downregulated *MYB33*, *MYB65*, or *MYB101* expression.
Stomata density on both abaxial (A) and adaxial (B) leaf surfaces in *Arabidopsis* mutant plants with downregulated level of *MYB33*, *MYB65*, or *MYB101* genes when compared to wild‐type plants. (A) Upper panel shows light micrographs of abaxial leaf surface. Each stomata is circled in red for visualization. Scale bar: 20 μm. Lower panel presents a table showing a comparison of the abaxial leaf stomata density in wild‐type and transgenic plants representing three independent SALK mutant lines exhibiting downregulation of *MYB33*, *MYB65*, and *MYB101* gene expression, respectively. Blue bar: wild‐type plants. Colored bars represent selected mutant lines. (B) The same experiments as in the (A) panel performed for stomata density on the adaxial leaf surface of wild‐type and *myb33*, *myb65*, or *myb101* mutant plants. Upper panel scale bar: 50 μm. (C) Upper panel: trichome density on adaxial leaf surfaces in *Arabidopsis myb33*, *myb65*, and *myb101* mutant plants when compared to wild‐type plants. Each trichome is circled in red for visualization. Scale bar: 1 mm. Lower panel: a table showing a comparison of the adaxial leaf trichome density in wild‐type and transgenic plants representing three independent SALK mutant lines exhibiting downregulation of *MYB33*, *MYB65*, and *MYB101* gene expression. Blue bar: wild‐type plants. Colored bars represent selected mutant lines. Values are shown as the mean ± sd (*n* = 10). Mann–Whitney test, *p* value: *p = 0.05; **p = 0.01; ***p = 0.001.

**FIGURE S3** Cuticle ultrastructure is differently affected by the downregulation of *MYB33*, *MYB65*, and *MYB101* gene expression levels.
Adaxial cuticle ultrastructure presented on TEM micrographs (upper panel). Arrows point to the cuticle layer. Scale bar: 50 nm. Lower panel: graphs showing measurements of cuticle thickness in each mutant in comparison to WT. Mann–Whitney test, *p* value: *p = 0.05; **p = 0.01; ***p = 0.001.

**FIGURE S4** Confirmation of mRNA and protein expression of At*MYB33*, At*MYB65*, At*MYB101*, and St*MYB33* transgenes in *Arabidopsis* mutant plants.
(A) Schematic representation of changes in microRNA159 recognition sites in mRNA sequences of studied *MYB* genes. (B) Agarose gel electrophoresis of RT‐PCR products of *AtMYB33*, *AtMYB65*, *AtMYB101*, and *StMYB33* cDNAs in a number of transgenic lines compared to WT plants. Arrows point to the obtained proper products; lower panels show expression of actin cDNA as a control. WT, wild‐type; M, GeneRuler 100 bp + DNA Ladder; −, negative control. (C) Western blots showing FLAG‐tagged MYB proteins expressed in selected *Arabidopsis* transgenic lines (upper panels) in comparison to WT; antiactin antibodies were used as a control (lower panels). M, protein weight marker.

**FIGURE S5** *AtMYB101* OE heterozygote plants siliques reveal problems with fertilization and embryo development of homozygous *AtMYB101*OE seeds.
Binocular graphs of *Arabidopsis* green siliques, cut perpendicularly on one side of the replum, with valves flattened on the both sides of the septum. Three lines: 2‐2‐2, 2‐11‐2, and 2‐11‐4 of plants with overexpression of *AtMYB101*, show spots where seeds did not develop and/or the development was arrested, while in WT plants septum has complete set of the seeds. This result indicates that two copies of *AtMYB101* transgene are lethal for the plants at the embryo developmental stage. Scale bar: 2 mm.

**FIGURE S6** Stomata and trichomes density on the surfaces of *Arabidopsis* leaves are differentially affected in the *Arabidopsis* mutant plants overexpressing *AtMYB33*, *AtMYB65*, *AtMYB101*, or *StMYB33* transgenes.
(A) Tables show a comparison of the abaxial (upper panel) or adaxial (lower panel) leaf stomata density in wild‐type and transgenic plants representing three independent transgenic lines exhibiting overexpression of *MYB33, MYB65*, and *MYB101* gene expression, respectively. Blue bar: wild‐type plants. Colored bars represent selected mutant lines. (B) Tables show trichome density on adaxial leaf surfaces in the same *Arabidopsis* mutant plants as in (A). Values are shown as the mean ± sd (*n* = 9). Mann–Whitney test, *p* value: *p = 0.05; **p = 0.01; ***p = 0.001.

**FIGURE S7** Cuticle is thinner in the transgenic *Arabidopsis* plants overexpressing *MYB33*, *MYB65*, and *MYB101* genes.
(A) Adaxial cuticle ultrastructure presented on TEM micrographs. Arrows point to the cuticle layer. Scale bar: 50 nm. (B) Graphs show measurements of cuticle thickness in each mutant in comparison to WT. Mann–Whitney test, *p* value: *p = 0.05; **p = 0.01; ***p = 0.001.

**FIGURE S8** Confirmation of mRNA expression of At*MYB33*, At*MYB65*, At*MYB101*, St*MYB33* and *StMYB65* transgenes in potato mutant plants.
Agarose gel electrophoresis showing RT‐PCR products of resistance to hygromycin gene mRNA (A) and fragments of over‐expressed cDNAs, respectively (B) in individual transgenic lines of each OE construct, comparing to WT Désireé plants. Numbers represent individual transgenic lines. Arrows point to the obtained proper products. WT, wild‐type; Deriree; M, Low Range GeneRuler DNA Ladder; −, negative control.

**FIGURE S9** Potato transgenic plants overexpressing *AthMYB33*, *AthMYB65*, *AthMYB101*, *StMYB33*, and *StMYB65*, respectively grown in vitro and in pots.
(A) In vitro grown potato plants with over expression of *AtMYB33*, AtMYB65, and *AtMYB101* genes do not show phenotypic differences in comparison to wild‐type plants. Only the plants with over expression of *StMYB65* show strong dwarfing phenotype. Scale bars: 2.5 cm. (B) Overexpression of potato MYB TFs strongly affects potato phenotype. Comparison of wild‐type potato plant with *StMYB33* OE and *StMYB65* OE. Scale bar: 15 cm.

**FIGURE S10** Stomata and trichomes density on the surfaces of leaves are differentially affected in the potato mutant plants overexpressing *AtMYB33*, *AtMYB65*, *AtMYB101*, or *StMYB33* transgenes.
(A) Tables show a comparison of the abaxial (A) or adaxial (B) leaf stomata density in wild‐type and transgenic plants representing three independent transgenic lines exhibiting overexpression of *MYB33*, *MYB65*, *MYB101*, and *StMYB33* genes. Blue bar: wild‐type plants. Colored bars represent selected mutant lines. (C, D) Tables show trichome density on abaxial (C) and adaxial (D) leaf surfaces in the same potato mutant plants as in (A). Values are shown as the mean ± sd (*n* = 9). *p* value: *p = 0.05; **p = 0.01; ***p = 0.001; Mann–Whitney test.

**FIGURE S11** *Solanum tuberosum* var. Désireé transgenic plants with overexpression of Ath*MYB33*, Ath*MYB65*, *AthMYB101* genes and St*MYB33* gene are resistant to drought.
(A) Upper panel presents transgenic potato plant overexpressing *AtMYB33* gene (line AtMYB33‐4 OE) compared with WT plant. Plants are shown in Day 11 (D11) after water cessation. Lower panel: RWC measurements in leaves from WT and transgenic *AtMYB33* OE (A33) plants after drought stress. The RWC value of the control (Désireé: 0.80) is the value of difference between its water content on the D0 and D11. Blue bars: WT plants, other colored bars represent potato plants from independent transgenic lines overexpressing *AtMYB33*. (B–D) show the same data obtained for potato transgenic plants overexpressing *AtMYB65* (B), *AtMYB101* (C), and *StMYB33* (D), respectively. Figure descriptions – as in the (A) panels. (A, B, C, and D) RWC data shown as the mean ± sd of *n* = 3 independent experiments. Mann–Whitney test, *p* value: *p = 0.05; **p = 0.01; ***p = 0.001. Scale bar: 15 cm.

**APPENDIX S1** Supplemental data

## Data Availability

Manuscript was deposited in bioRxiv DOI: https://doi.org/10.1101/2021.04.07.438025.
