# Supplementary figures and images for: The MYB33, MYB65, and MYB101 transcription factors affect Arabidopsis and potato responses to drought by regulating the ABA signaling pathway
Source: Physiol Plant. 2022 Sep 26;174(5):e13775. doi: 10.1111/ppl.13775 (PMC9828139; doi:10.1111/ppl.13775)

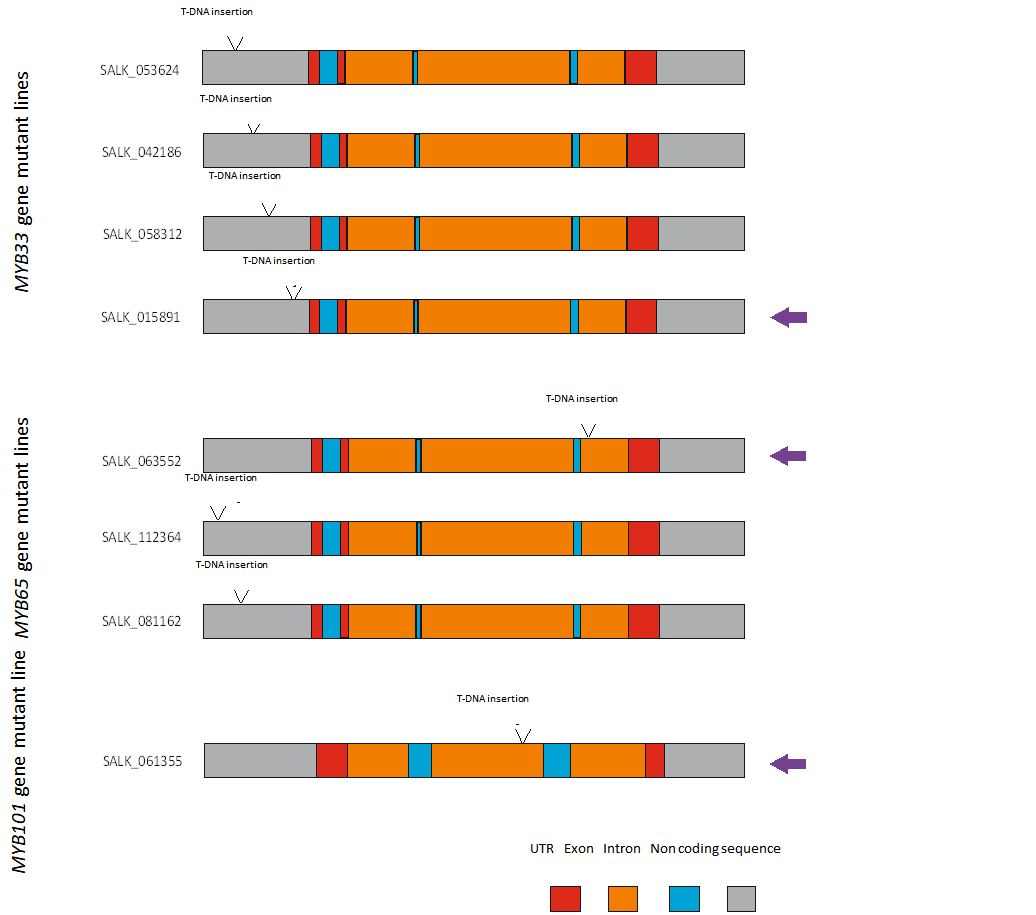

Supplement: Supplementary file 1 — FIGURE S1 Arabidopsis MYB33, MYB65, and MYB101 gene T‐DNA insertion lines for analysis of gene knock‐down or downregulation. Schematic graphs represent MYB33, MYB65, and MYB101 gene structures. Gray color represents gene promotor and 3′ flanking region, red color represents 5′ and 3’ UTRs, orange color represents exons, and blue color represents intron. SALK numbers are given at the left side of each gene harboring T‐DNA insertion. Localization of T‐DNA insertion is marked in the each gene structure. Violet arrows point T‐DNA insertion lines for further analysis. [file PPL-174-0-s008.tif]

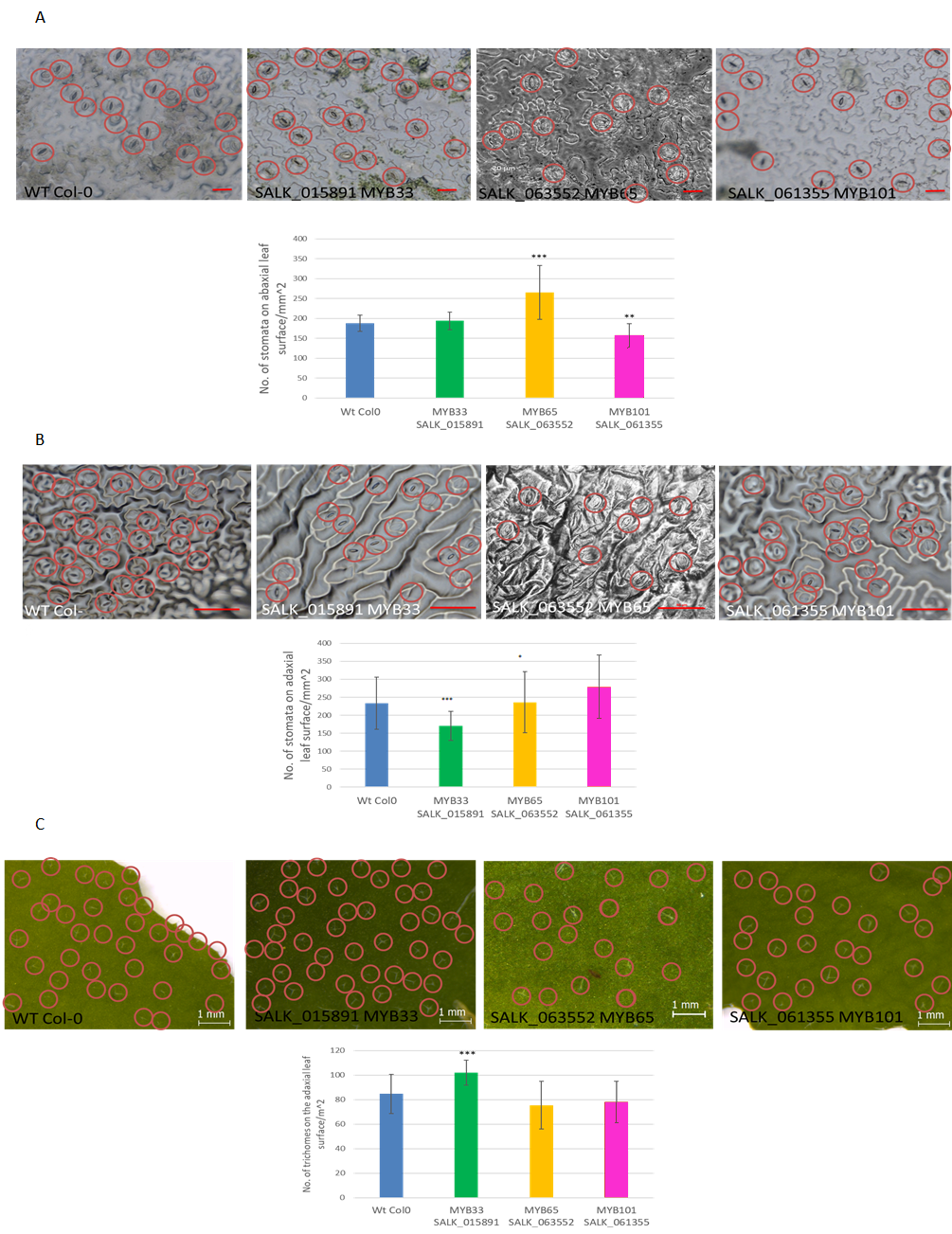

Supplement: Supplementary file 2 — FIGURE S2 Stomata and trichomes density on the surfaces of Arabidopsis leaves are differentially affected in the Arabidopsis mutant plants with the downregulated MYB33, MYB65, or MYB101 expression. Stomata density on both abaxial (A) and adaxial (B) leaf surfaces in Arabidopsis mutant plants with downregulated level of MYB33, MYB65, or MYB101 genes when compared to wild‐type plants. (A) Upper panel shows light micrographs of abaxial leaf surface. Each stomata is circled in red for visualization. Scale bar: 20 μm. Lower panel presents a table showing a comparison of the abaxial leaf stomata density in wild‐type and transgenic plants representing three independent SALK mutant lines exhibiting downregulation of MYB33, MYB65, and MYB101 gene expression, respectively. Blue bar: wild‐type plants. Colored bars represent selected mutant lines. (B) The same experiments as in the (A) panel performed for stomata density on the adaxial leaf surface of wild‐type and myb33, myb65, or myb101 mutant plants. Upper panel scale bar: 50 μm. (C) Upper panel: trichome density on adaxial leaf surfaces in Arabidopsis myb33, myb65, and myb101 mutant plants when compared to wild‐type plants. Each trichome is circled in red for visualization. Scale bar: 1 mm. Lower panel: a table showing a comparison of the adaxial leaf trichome density in wild‐type and transgenic plants representing three independent SALK mutant lines exhibiting downregulation of MYB33, MYB65, and MYB101 gene expression. Blue bar: wild‐type plants. Colored bars represent selected mutant lines. Values are shown as the mean ± sd (n = 10). Mann–Whitney test, p value: *p = 0.05; **p = 0.01; ***p = 0.001. [file PPL-174-0-s005.tiff]

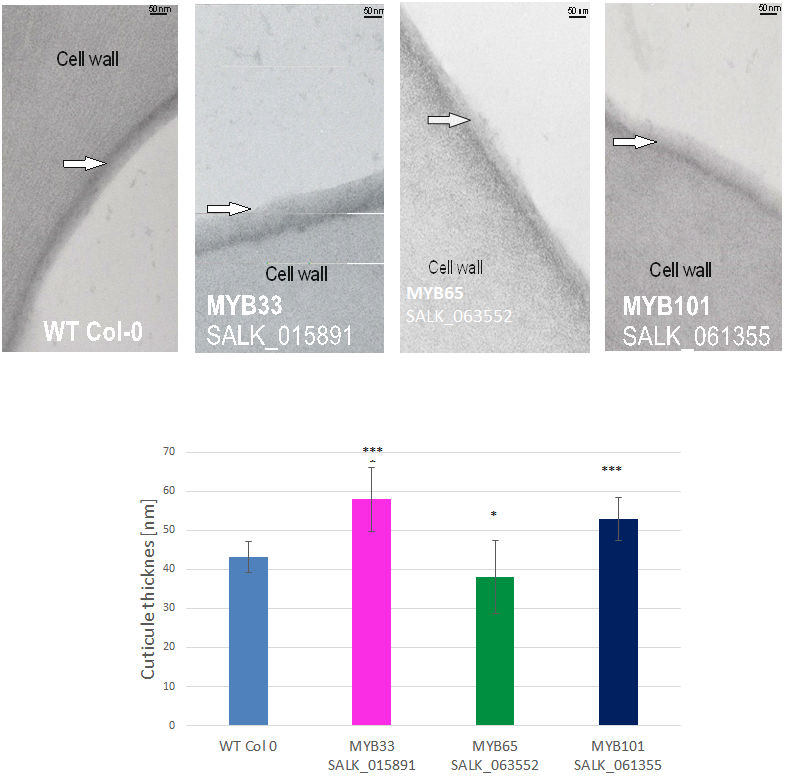

Supplement: Supplementary file 3 — FIGURE S3 Cuticle ultrastructure is differently affected by the downregulation of MYB33, MYB65, and MYB101 gene expression levels. Adaxial cuticle ultrastructure presented on TEM micrographs (upper panel). Arrows point to the cuticle layer. Scale bar: 50 nm. Lower panel: graphs showing measurements of cuticle thickness in each mutant in comparison to WT. Mann–Whitney test, p value: *p = 0.05; **p = 0.01; ***p = 0.001. [file PPL-174-0-s006.tif]

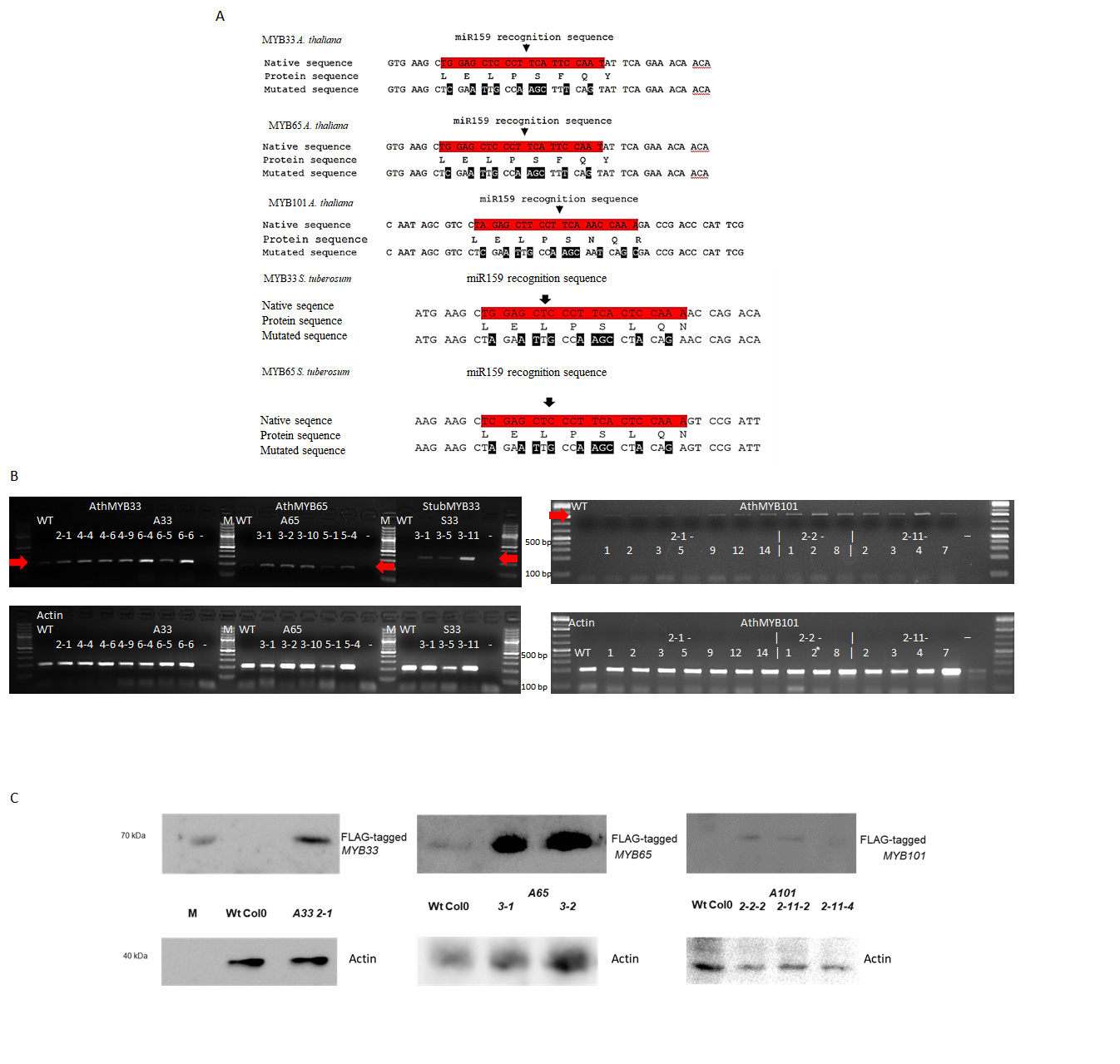

Supplement: Supplementary file 4 — FIGURE S4 Confirmation of mRNA and protein expression of AtMYB33, AtMYB65, AtMYB101, and StMYB33 transgenes in Arabidopsis mutant plants. (A) Schematic representation of changes in microRNA159 recognition sites in mRNA sequences of studied MYB genes. (B) Agarose gel electrophoresis of RT‐PCR products of AtMYB33, AtMYB65, AtMYB101, and StMYB33 cDNAs in a number of transgenic lines compared to WT plants. Arrows point to the obtained proper products; lower panels show expression of actin cDNA as a control. WT, wild‐type; M, GeneRuler 100 bp + DNA Ladder; −, negative control. (C) Western blots showing FLAG‐tagged MYB proteins expressed in selected Arabidopsis transgenic lines (upper panels) in comparison to WT; antiactin antibodies were used as a control (lower panels). M, protein weight marker. [file PPL-174-0-s002.tif]

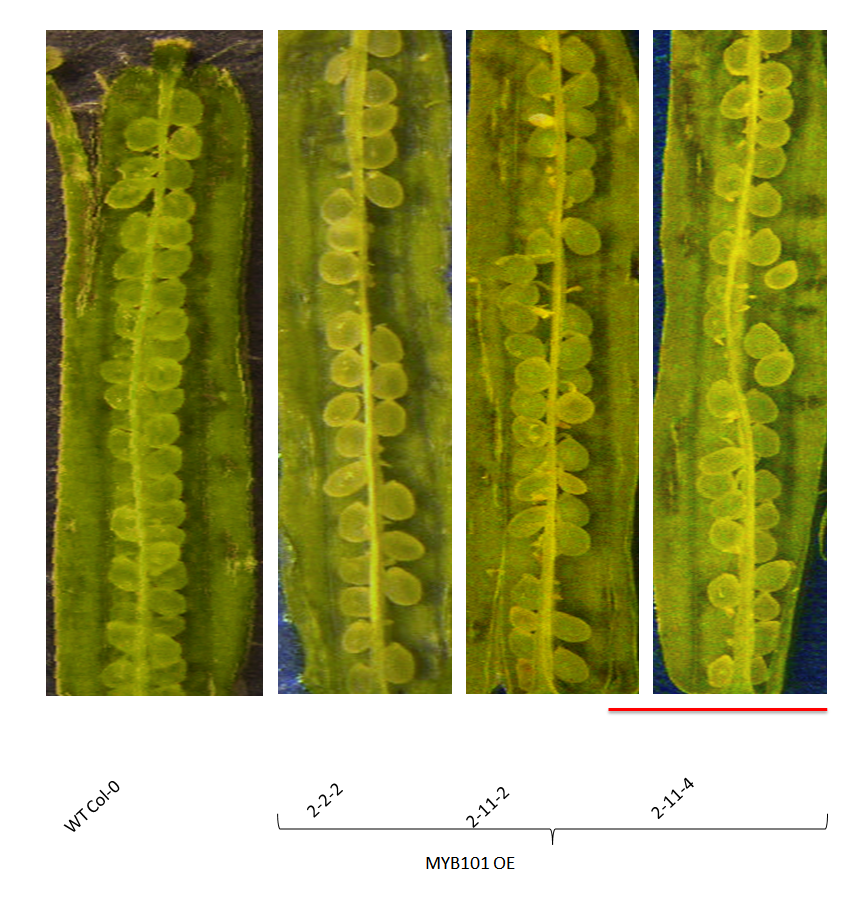

Supplement: Supplementary file 5 — FIGURE S5 AtMYB101 OE heterozygote plants siliques reveal problems with fertilization and embryo development of homozygous AtMYB101OE seeds. Binocular graphs of Arabidopsis green siliques, cut perpendicularly on one side of the replum, with valves flattened on the both sides of the septum. Three lines: 2‐2‐2, 2‐11‐2, and 2‐11‐4 of plants with overexpression of AtMYB101, show spots where seeds did not develop and/or the development was arrested, while in WT plants septum has complete set of the seeds. This result indicates that two copies of AtMYB101 transgene are lethal for the plants at the embryo developmental stage. Scale bar: 2 mm. [file PPL-174-0-s010.tif]

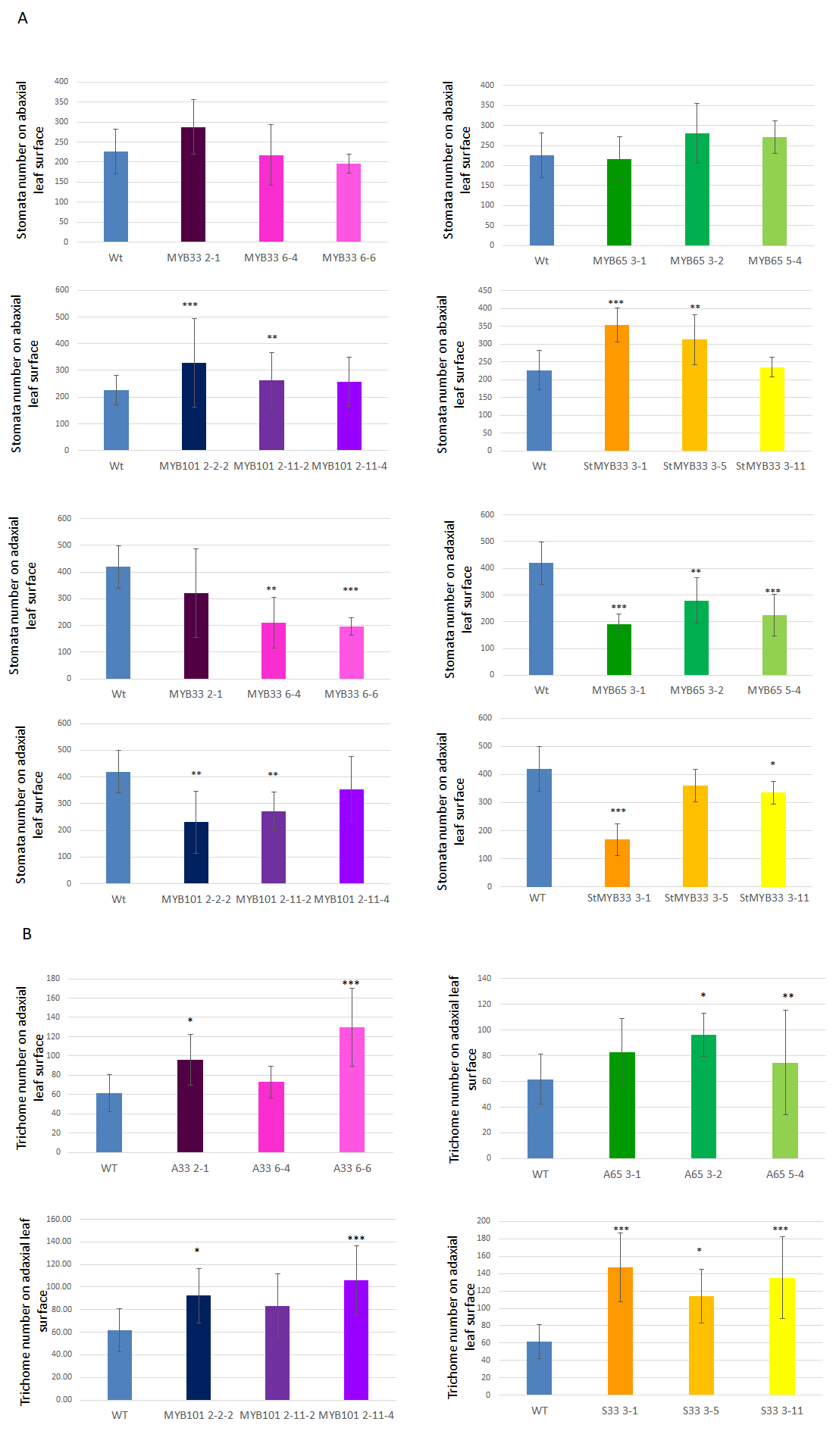

Supplement: Supplementary file 6 — FIGURE S6 Stomata and trichomes density on the surfaces of Arabidopsis leaves are differentially affected in the Arabidopsis mutant plants overexpressing AtMYB33, AtMYB65, AtMYB101, or StMYB33 transgenes. (A) Tables show a comparison of the abaxial (upper panel) or adaxial (lower panel) leaf stomata density in wild‐type and transgenic plants representing three independent transgenic lines exhibiting overexpression of MYB33, MYB65, and MYB101 gene expression, respectively. Blue bar: wild‐type plants. Colored bars represent selected mutant lines. (B) Tables show trichome density on adaxial leaf surfaces in the same Arabidopsis mutant plants as in (A). Values are shown as the mean ± sd (n = 9). Mann–Whitney test, p value: *p = 0.05; **p = 0.01; ***p = 0.001. [file PPL-174-0-s011.tif]

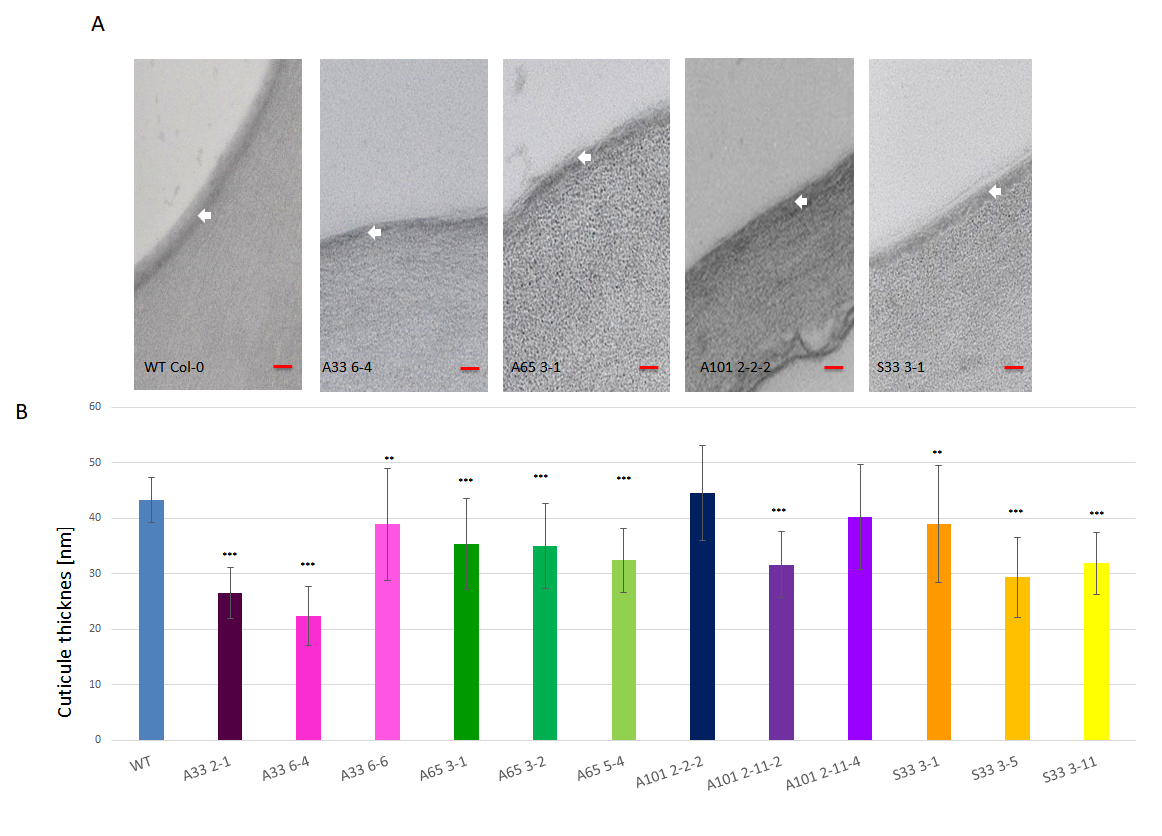

Supplement: Supplementary file 7 — FIGURE S7 Cuticle is thinner in the transgenic Arabidopsis plants overexpressing MYB33, MYB65, and MYB101 genes. (A) Adaxial cuticle ultrastructure presented on TEM micrographs. Arrows point to the cuticle layer. Scale bar: 50 nm. (B) Graphs show measurements of cuticle thickness in each mutant in comparison to WT. Mann–Whitney test, p value: *p = 0.05; **p = 0.01; ***p = 0.001. [file PPL-174-0-s001.tif]

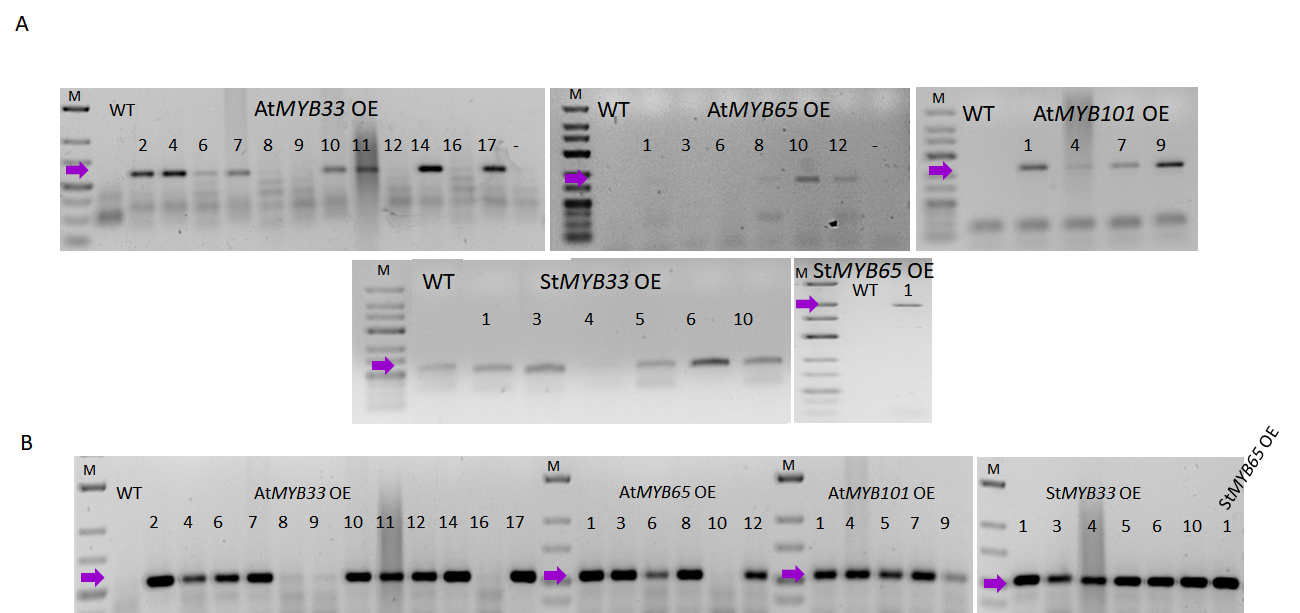

Supplement: Supplementary file 8 — FIGURE S8 Confirmation of mRNA expression of AtMYB33, AtMYB65, AtMYB101, StMYB33 and StMYB65 transgenes in potato mutant plants. Agarose gel electrophoresis showing RT‐PCR products of resistance to hygromycin gene mRNA (A) and fragments of over‐expressed cDNAs, respectively (B) in individual transgenic lines of each OE construct, comparing to WT Désireé plants. Numbers represent individual transgenic lines. Arrows point to the obtained proper products. WT, wild‐type; Deriree; M, Low Range GeneRuler DNA Ladder; −, negative control. [file PPL-174-0-s004.tif]

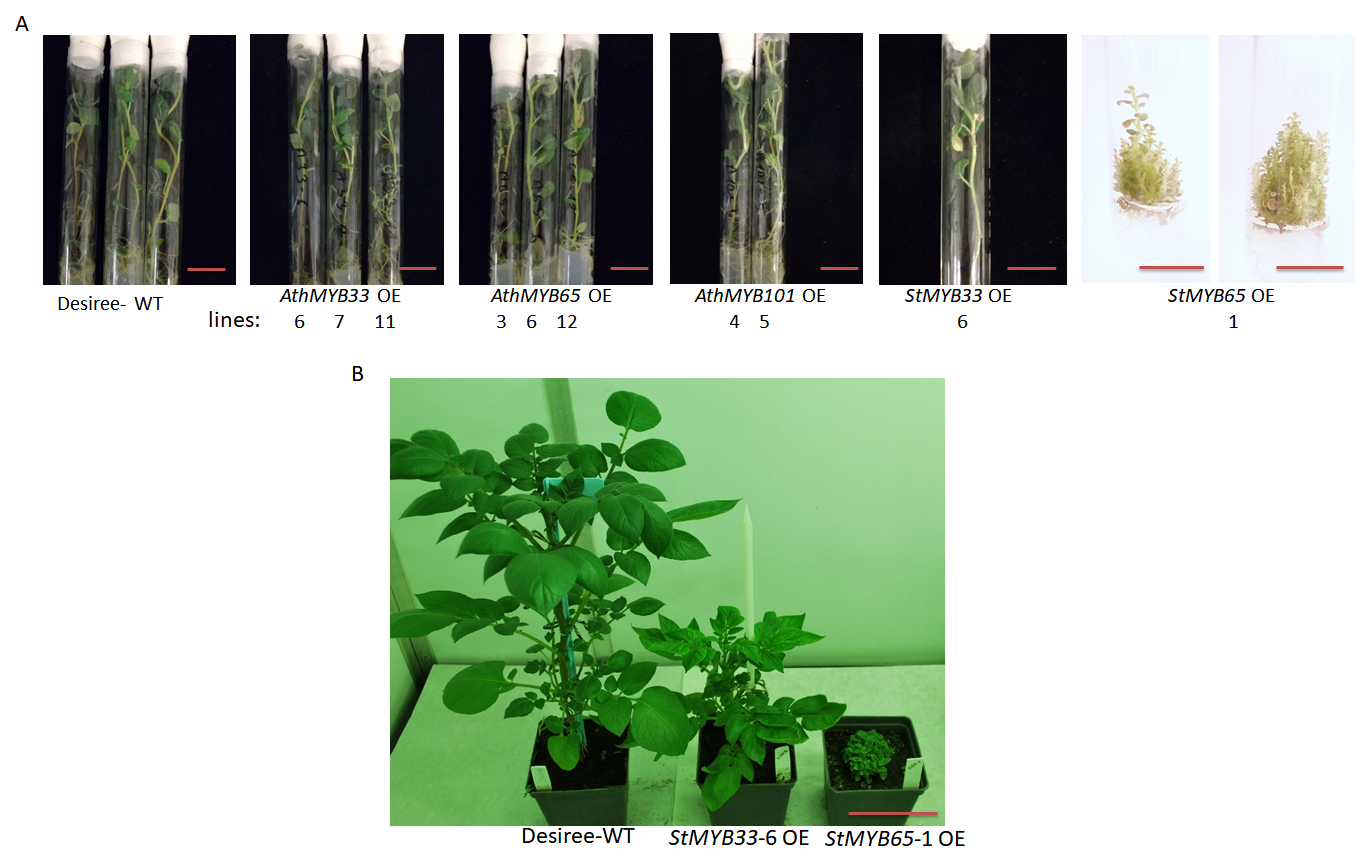

Supplement: Supplementary file 9 — FIGURE S9 Potato transgenic plants overexpressing AthMYB33, AthMYB65, AthMYB101, StMYB33, and StMYB65, respectively grown in vitro and in pots. (A) In vitro grown potato plants with over expression of AtMYB33, AtMYB65, and AtMYB101 genes do not show phenotypic differences in comparison to wild‐type plants. Only the plants with over expression of StMYB65 show strong dwarfing phenotype. Scale bars: 2.5 cm. (B) Overexpression of potato MYB TFs strongly affects potato phenotype. Comparison of wild‐type potato plant with StMYB33 OE and StMYB65 OE. Scale bar: 15 cm. [file PPL-174-0-s009.tif]

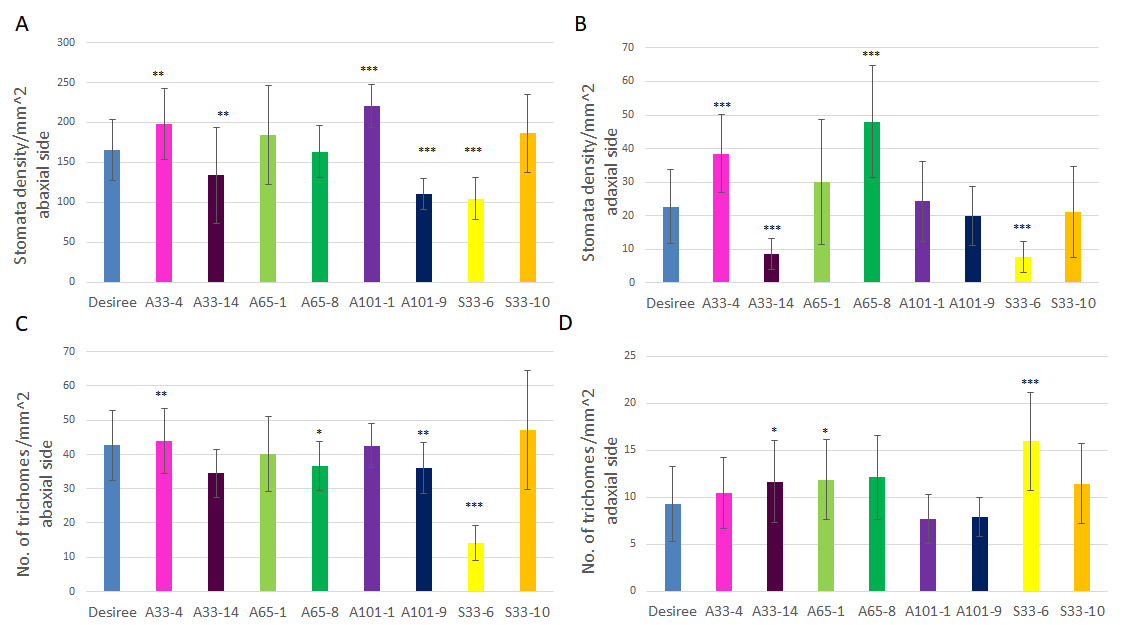

Supplement: Supplementary file 10 — FIGURE S10 Stomata and trichomes density on the surfaces of leaves are differentially affected in the potato mutant plants overexpressing AtMYB33, AtMYB65, AtMYB101, or StMYB33 transgenes. (A) Tables show a comparison of the abaxial (A) or adaxial (B) leaf stomata density in wild‐type and transgenic plants representing three independent transgenic lines exhibiting overexpression of MYB33, MYB65, MYB101, and StMYB33 genes. Blue bar: wild‐type plants. Colored bars represent selected mutant lines. (C, D) Tables show trichome density on abaxial (C) and adaxial (D) leaf surfaces in the same potato mutant plants as in (A). Values are shown as the mean ± sd (n = 9). p value: *p = 0.05; **p = 0.01; ***p = 0.001; Mann–Whitney test. [file PPL-174-0-s012.tif]

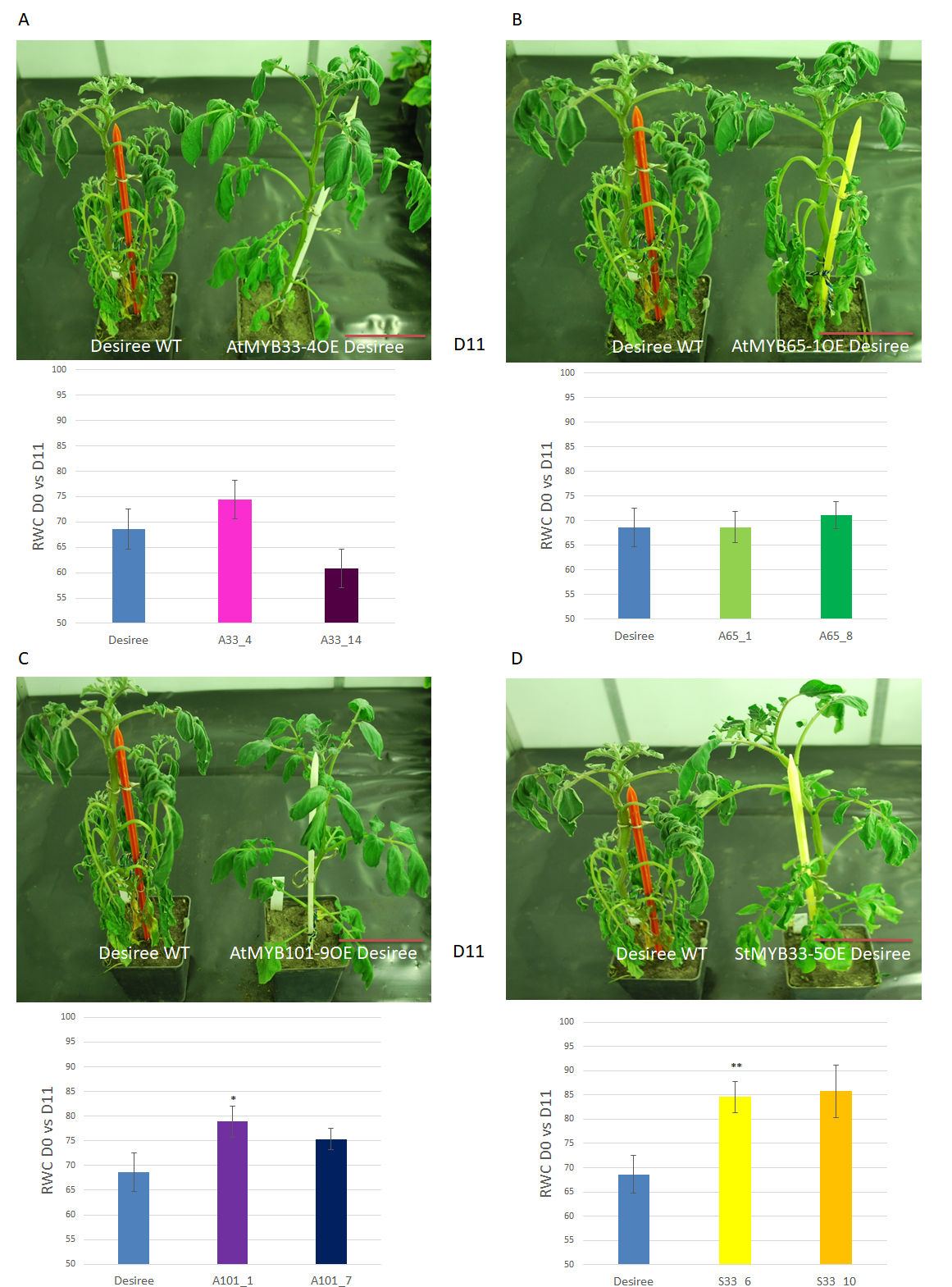

Supplement: Supplementary file 11 — FIGURE S11 Solanum tuberosum var. Désireé transgenic plants with overexpression of AthMYB33, AthMYB65, AthMYB101 genes and StMYB33 gene are resistant to drought. (A) Upper panel presents transgenic potato plant overexpressing AtMYB33 gene (line AtMYB33‐4 OE) compared with WT plant. Plants are shown in Day 11 (D11) after water cessation. Lower panel: RWC measurements in leaves from WT and transgenic AtMYB33 OE (A33) plants after drought stress. The RWC value of the control (Désireé: 0.80) is the value of difference between its water content on the D0 and D11. Blue bars: WT plants, other colored bars represent potato plants from independent transgenic lines overexpressing AtMYB33. (B–D) show the same data obtained for potato transgenic plants overexpressing AtMYB65 (B), AtMYB101 (C), and StMYB33 (D), respectively. Figure descriptions – as in the (A) panels. (A, B, C, and D) RWC data shown as the mean ± sd of n = 3 independent experiments. Mann–Whitney test, p value: *p = 0.05; **p = 0.01; ***p = 0.001. Scale bar: 15 cm. [file PPL-174-0-s003.tif]
