# Supplementary material for: The MYB33, MYB65, and MYB101 transcription factors affect Arabidopsis and potato responses to drought by regulating the ABA signaling pathway
Source: Physiol Plant. 2022 Sep 26;174(5):e13775. doi: 10.1111/ppl.13775 (PMC9828139; doi:10.1111/ppl.13775)
Supplement: Supplementary file 12 — APPENDIX S1 Supplemental data [file PPL-174-0-s007.docx]

**Suplemental data**

**Protein extraction and Western blots**

Five hundred microliters of protein extraction buffer containing 200 mM Tris–HCl (pH 7.5), 250 mM NaCl, 25 mM EDTA (pH 8.0) and 0.5% SDS was added to frozen, ground plant material in an Eppendorf tube, which was subsequently vortexed at 1000-1100 rpm for 30 min at 4°C. The samples were then centrifuged at 18000 g for 30 min at 4°C. The supernatant was collected, separated into 5 tubes (100 μl per tube) and either further used or stored at -20°C for a short period of time. The amount of protein in the extracts were assessed by the Bradford method using Bio-Rad Protein Assay Dye Reagent Concentrate (Bio-Rad). Protein electrophoresis in 10% or 12% PAA gels with SDS in 1x Laemmli buffer was carried out, after which the proteins were transferred to Immobilon P membranes (Merck Millipore). After blocking the membrane with 5% skimmed milk in TBS-T buffer solution for 1h at RT or overnight at 4°C, the membrane was incubated with antibodies (primary anti-actin at a 1:5000 ratio for 1.5h at RT and anti-FLAG conjugated with horseradish peroxidase at a 1:1000 ratio for 3h at RT). The membrane containing actin was then washed in TBS-T buffer 3 times for 10 min and subsequently incubated in solution containing secondary antibodies (anti-mouse at a 1:10000 ratio for 1h at RT). After washing both parts of the membrane (as before, in TBS buffer), freshly mixed equal volumes of ECL Western Blotting Detection Reagents A and B (Amersham) were poured onto the membranes, which were subsequently incubated for 5 min and then removed. Detection of chemiluminescence was conducted via a G:Box apparatus and GeneSys (Syngene) software.
